# The Role of Isoflavones in the Prevention of Breast Cancer and Prostate Cancer

**DOI:** 10.3390/antiox12020368

**Published:** 2023-02-03

**Authors:** Tomislav Pejčić, Milica Zeković, Uroš Bumbaširević, Milica Kalaba, Irena Vovk, Maja Bensa, Lazar Popović, Živoslav Tešić

**Affiliations:** 1 Faculty of Medicine, University of Belgrade, dr Subotića 8, 11000 Belgrade, Serbia; 2 Clinic of Urology, University Clinical Center of Serbia, Pasterova 2, 11000 Belgrade, Serbia; 3 Centre of Research Excellence in Nutrition and Metabolism, Institute for Medical Research, National Institute of Republic of Serbia, University of Belgrade, Tadeusa Koscuska 1, 11000 Belgrade, Serbia; 4 Institute of General and Physical Chemistry, Studentski trg 12-16, 11158 Belgrade, Serbia; 5 Laboratory for Food Chemistry, National Institute of Chemistry, Hajdrihova 19, 1000 Ljubljana, Slovenia; 6 Faculty of Health Sciences, University of Ljubljana, Zdravstvena pot 5, 1000 Ljubljana, Slovenia; 7 Department of Medical Oncology, Oncology Institute of Vojvodina, Put Doktora Goldmana 4, 21204 Sremska Kamenica, Serbia; 8 Faculty of Medicine Novi Sad, University of Novi Sad, Hajduk Veljkova 3, 21000 Novi Sad, Serbia; 9 Faculty of Chemistry, University of Belgrade, Studentski trg 12-16, 11158 Belgrade, Serbia

**Keywords:** isoflavones, breast cancer, prostate cancer

## Abstract

This narrative review summarizes epidemiological studies on breast cancer and prostate cancer with an overview of their global incidence distribution to investigate the relationship between these diseases and diet. The biological properties, mechanisms of action, and available data supporting the potential role of isoflavones in the prevention of breast cancer and prostate cancer are discussed. Studies evaluating the effects of isoflavones in tissue cultures of normal and malignant breast and prostate cells, as well as the current body of research regarding the effects of isoflavones attained through multiple modifications of cellular molecular signaling pathways and control of oxidative stress, are summarized. Furthermore, this review compiles literature sources reporting on the following: (1) levels of estrogen in breast and prostate tissue; (2) levels of isoflavones in the normal and malignant tissue of these organs in European and Asian populations; (3) average concentrations of isoflavones in the secretion of these organs (milk and semen). Finally, particular emphasis is placed on studies investigating the effect of isoflavones on tissues via estrogen receptors (ER).

## 1. Introduction

At least three facts support the thesis that the Western dietary pattern featuring high consumption of processed red meat and fat and limited intake of plant-based foods may be associated with the increased risk of breast cancer and prostate cancer. First, these oncopathologies are most common in geographical areas with predominant Western-style nutrition and quite rare in parts of Asia where soy and unrefined plant foods are the main dietary components. Second, extensive literature suggests markedly low incidence rates of breast cancer and prostate cancer among great apes, who are herbivores and the closest evolutionary cousins of modern humans. Third, Homo sapiens was obligatory herbivore until it began to introduce meat into the diet. This significant change in evolutionary dietary trajectory is now hypothesized to be an important factor in the rising incidence of breast cancer and prostate cancer in humans [1,2]. Exploration of nutritional factors and diet-derived mediators is a rapidly growing area of anticancer research. Remarkable advancements in high-throughput genomic technologies, molecular epidemiology and biochemical techniques have paved the way for a more holistic and precise approach to deciphering the relationship between bioactive food compounds and cancer at the cellular, individual and population level [3].

The similarity between breast and prostate tissue is reflected in the presence of connective stroma and glandular secretory epithelium. Through constant interaction, the stroma and glandular epithelium control the secretory activity of these organs. Sex hormones, testosterone and estrogen, mostly activate stromal cells via the androgen receptor (AR) and two estrogen receptors (ER): ER-α and ER-β. The ER-α mediates stroma proliferation in both normal and malignant tissue, while the ER-β opposes the action of ER-α [4]. With functional features and structural elements correspondent to the superfamily of nuclear hormone receptors, ERs modulate the transcription of the specific target genes through complex genomic and non-genomic mechanisms and additional convergent cascades, thereby controlling a wide array of physiological functions in respective organ systems, including reproductive, cardiovascular, skeletal and central nervous systems [5]. Being encoded with distinct genes (ESR1 and ESR2), these receptor subtypes have diverse spatial and temporal tissue expression patterns and differential ligand specificities and regulate different physiological processes [6]. The variable absolute expression level and the context-specific variants-ratio are profoundly associated with their biological functions: the alpha subtype is reported to have a more significant role in the cell growth and differentiation in the uterus, prostate and mammary glands as well as the maintenance of metabolic and skeletal homeostasis, whereas the beta form dominantly affects the immune and central nervous systems and counteracts the ERα-induced cell proliferation in estrogen-sensitive tissues [7].

Isoflavones are an important subgroup of flavonoids, a large class of naturally occurring phenolic compounds with great structural and functional diversity. Aglycons genistein, daidzein and glycitein, and their glycosides genistin, daidzin and glycitin are considered to be the most important representatives of isoflavones. Major sources of isoflavones are soybeans and other legumes. In plants, isoflavones play a critical role in the formation of phytoalexins during defense against microbes, while in humans, due to their structural similarity to 17β-estradiol, isoflavones exert significant estrogenic activity [8,9]. In contrast with estradiol, which activates both ERs equally, isoflavones are more selective. For example, genistein, the most important isoflavone, has a substantially higher binding affinity for the ER-β isoform than for the ER-α isoform. Genistein competes with estradiol and acts as an ER-α antagonist as well as a potent ER-β agonist [10].

## 2. Polyphenols—Chemical Characteristics and Division

Polyphenols are plant secondary metabolites and are naturally present in different plant parts [11,12]. In plants, polyphenols have an important role as a part of the plant’s defense mechanisms against different pathogens. Polyphenols are present in the human diet since they can be found in different fruits [12,13,14,15,16,17] and vegetables [18], medicinal plants [19,20,21,22] as well as other products such as vines [15] and bee products [21,23,24,25]. Differences in the quantity of polyphenols and their bioavailability for humans depend on plant type, food product, etc.

**Figure 1:**
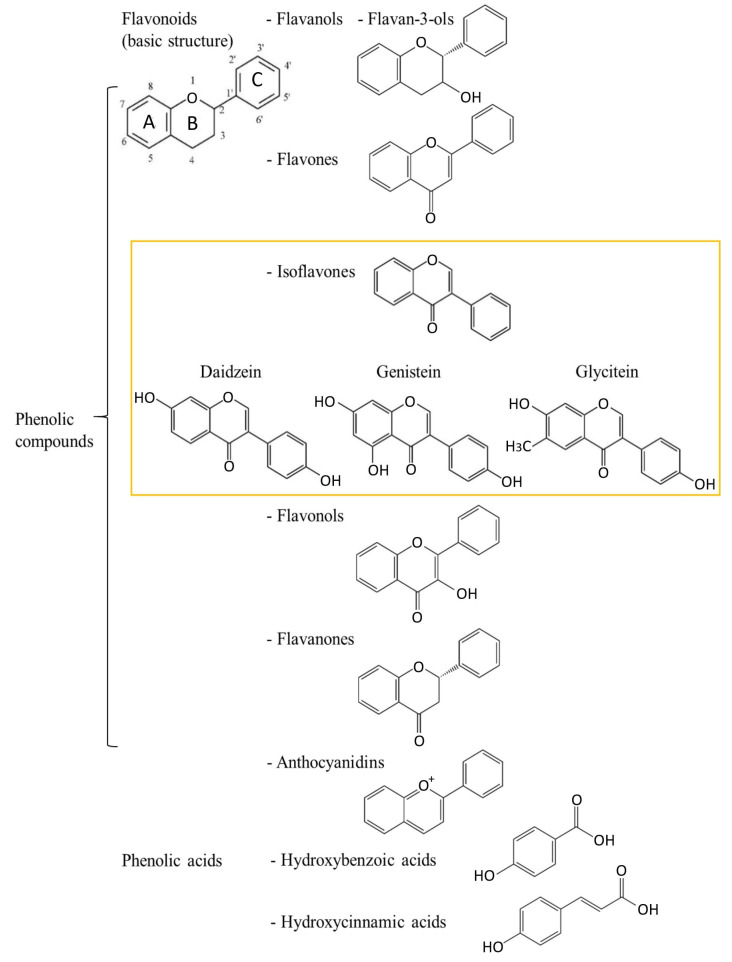
Schematic representation of the phenolic compounds and their chemical structures.

Polyphenols are a large group of structurally diverse compounds with a common characteristic of their chemical structures—an aromatic ring with at least one hydroxyl group. Polyphenols can be classified based on their chemical structure in different ways (Figure 1). One classification divides polyphenols into two large groups: flavonoids and non-flavonoids, which consist of more subgroups. Isoflavonoids, a group of flavonoids, are further divided into isoflavones and other groups.

Since polyphenols have different forms [16,26], their biological activity can be manifested in different ways according to the group to which they belong. Polyphenols can be considered to be valuable natural antioxidants that reduce free radicals through several mechanisms [26]. Free radicals—molecules with an unpaired electron(s)—can damage other biomolecules, create new radicals and adversely affect cell functionality, which is related to different metabolic processes and reactions in the human body. Even small concentrations of antioxidants can prevent or slow down oxidation, which results in cell protection [27]. An excess of free radicals, which often arises during various cell mutations and pathological processes, leads to oxidative stress, an important cause of diseases [28]. Antioxidant activity is important as antioxidative compounds activate the body’s defense system and affect radicals by converting them into other species.

Flavonoids, as good electron donors, are well-known antioxidants. They have the ability to scavenge free radicals by creating less reactive species. Antioxidant activity depends on the number and distribution of hydroxyl groups in their structures. The basic structural backbone formula is C_6_-C_3_-C_6_, where two rings, A and C, present chroman moiety and ring B phenyl moiety. Therefore, more OH^-^ groups bonded to the B ring achieve lower redox potential and, thus, more effective antioxidants. The saturation of the heterocyclic C ring stabilizes the radical and reduces the antioxidant activity of polyphenols (i.e., flavones and flavonols have higher antioxidant activity compared to flavans and flavanols). The subclass of isoflavones is characterized by ring B attached at C-3 (Figure 1).

The antioxidant activity of isoflavones was documented in the literature [8,29,30,31,32,33] and largely depends on their structure. Combinations of phenolic compounds with each other or with other molecules can have a synergistic effect [30,34]. Polyphenols are an important group of phytochemicals with a high potential for a positive impact on human health [35,36].

### 2.1. Isoflavones

**Figure 2:**
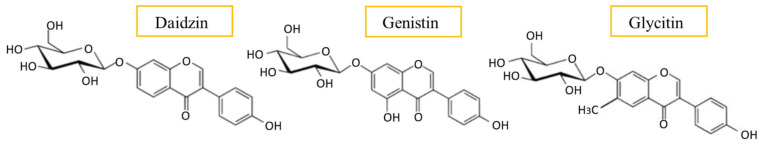
Structures of isoflavones glycosides: daidzin, genistin and glycitin.

Based on their structure, isoflavones are divided into three groups: (1) *O*-substituted derivatives (e.g., hydroxy, methoxy and methylenedioxy); (2) prenylated derivatives; (3) glycosylated derivatives [37]. For example, *O*-substituted isoflavones (and their glycosides, Figure 2) are daidzein (daidzin, Figure 2), genistein (genistin, Figure 2) and glycitein (glycitin, Figure 2) (Figure 1). Most often, isoflavones appear in the form of glycosides. In the human body, they are transformed into effective aglycones such as daidzein (7-hydroxy-3-(4-hydroxyphenyl)-4H-1-benzopyran-4-one) and genistein (5,7-dihydroxy-3-(4-hydroxyphenyl)-4H-1-benzopyran-4-one) and glycitein (7-hydroxy-3-(4-hydroxyphenyl)-6-methoxy-4H-1-benzopyran-4-one).

The USDA Database for the Isoflavone Content of Selected Foods provides an informative overview of isoflavone food sources [38]. Isoflavones represent polyphenols found in high content in soybean and other legumes as well as their products, with soybeans having higher contents of isoflavones compared to other legumes [8,37]. In soybean seeds, malonyl glycosides forms (malonyl daidzein and malonyl genistein) were present in a higher amount than aglycons (daidzein and genistein), while glucosides (daidzin and genistin) (Figure 2) were present in the lowest amount [29]. Glycitin (Figure 2), an *O*-methylated isoflavone glycoside (glycitein 7-*O-*glucoside), was also found in soy [39].

The content of isoflavones differs through the products and regions [38]. Daily consumption [40] in Asian countries is much higher than in other countries [8,41]. The consumption of food containing isoflavones is related to the traditional foods of countries such as China, Japan and South Korea. Higher dietary intake of isoflavones can be related to health benefits due to the biological activity of isoflavones [8,30].

#### Health-Related Properties of Isoflavones

Isoflavones are known to be beneficial for human health due to their many functional characteristics. Isoflavones are a type of phytoestrogens, which are estrogen-like non-steroid substances found in plants [8]. Isoflavones represent phytochemicals with identical physiological activity as estrogens, becoming physiologically active through the intestinal microflora [8,9]. For example, S-equol, a metabolite of daidzein and genistein, has a strong antioxidative effect [8], effects on cardiometabolic risk factors [42]), high estrogenic activity, as well as the highest antioxidant activity among isoflavones, with an effect on carcinoma cells. Levels of S-equol > 5–10 ng/mL have been associated as a therapeutic agent with a positive effect against prostate cancer and other health benefits [31,43]. Although isoflavone aglycones have better bioavailability and bioactivity than their glycosides [44], hydrolysis of glycosides to aglycones before consumption of a nonfermented soy drink did not enhance the bioavailability of isoflavones in postmenopausal women [45]. The structural similarity with estrogen indicates the possible biological effects of isoflavones and their potential for cardiovascular and hormone replacement therapy in postmenopausal women [8,40]. Other research has shown great interest in the health benefits of the association of S-equol and the gut microbial community shown by improvement in cardiometabolic risk factors [42]. Furthermore, the importance of isoflavones is reflected in investigations of their influence on cancer [8,31,32,37,41], described in detail in Section 3 and Section 4. The usefulness of polyphenols synergism, their relationship with other phytochemicals, as well as food intake, are of critical importance to understanding the overall impact of isoflavones.

## 3. Breast Cancer and Isoflavones

### 3.1. Breast Cancer—Epidemiology and Risk Factors

With extraordinary heterogeneity in terms of occurrence rates, clinical presentation, available preventive and curative interventions, and therapeutic response, the overall magnitude of the public health burden related to cancer incidence and mortality is substantially increasing worldwide. The increasing cancer occurrence and mortality are the results of the cumulative effects of aging and the growth of the global population, as well as the transition of distribution and prevalence of major risk factors. Breast cancer is the most frequently occurring invasive cancer in the female population. With an annual estimation of 2.3 million new cases based on the Global Cancer Statistics—GLOBOCAN report for 2020, breast cancer surpassed lung cancer as the most commonly diagnosed malignancy accounting for 11.7% of total cases. Furthermore, breast cancer is considered to be the leading cause of cancer death among women and ranks fifth in combined-sex mortality estimates [46]. There is prominent geographic variability in breast cancer registry data. Although the age-standardized incidence rates are approximately 88% higher in transitioned countries (i.e., Australia, New Zealand, and other countries in North America, Western and Northern Europe) compared to transitioning countries (55.9 versus 29.7 per 100,000, respectively), mortality rates are 19% higher in regions with a lower level of socioeconomic development [46]. These patterns may be attributed to both variances in exposure to risk factors and to worldwide disparities across the continuum of healthcare [47]. Furthermore, global temporal-trend figures show that incidence rates have been increasing in virtually all regions over the last three decades. Although overall lethality indices continuously decline, case-fatality proportions remain markedly higher in lower-income countries, particularly in Africa and among small island nations in Melanesia, Micronesia, Polynesia and the Caribbean [48,49]. Current patterns in breast cancer incidence and survival trends are affected by: alterations in environmental and individual-level variables and risk factors, public health determinants (such as widespread mammography screening programs), and general advancements in diagnostic and treatment infrastructure [50,51]. Pronounced differences in mortality-to-incidence ratios warrant concern and an urgent need for methodical and harmonized dissemination and implementation of evidence-based, contextually suitable policies and interventions targeting awareness, education, risk identification, and early detection, as well as reinforcement of equitable resources and capacities for high-quality multimodal treatment and survivorship care [52,53]. Nevertheless, the rising incidence in developing countries in Africa, South America, and Asia, as well as in certain affluent Asian countries, where rates are traditionally low, is associated with the so-called “Westernization” phenomenon. This term refers to dramatic changes in sociocultural circumstances, economic development, urbanization, and demographic and workforce shifts that are accompanied by behavioral and lifestyle modifications. Similar incidence trend variations were observed in migration studies. Sedentary culture with less physical activity, increased prevalence of obesity, nutritional transition towards high-fat and (ultra) processed foods, postponement of childbirth and lower parity, and accessibility to hormonal birth control and replacement therapy are all contributing to the convergence towards the industrialized Western countries breast cancer risk-profile and the narrowing of the international gap between historically low and high morbidity areas [8,49].

Having a remarkably heterogeneous oncopathology, breast cancer comprises a wide spectrum of tumors with a high level of diversity in molecular alterations, cellular composition, histological features, clinical presentation, metastatic potential and dissemination patterns, treatment sensitivity and patient outcomes [54]. The classification of breast cancer is challenging due to the versatility of cancer cell phenotypes and their perplexing interaction with a plethora of molecular and cellular mediators of both tumoral and microenvironmental plasticity. The complexity of inter and intra-tumor variability is only partially epitomized by routinely applied principal clinical parameters (patient age, lymph node involvement, neoplasm size, tumor stage and grading) and pathobiological markers such as estrogen receptors (ER), progesterone receptors (PR), and human epidermal growth factor receptor 2 (HER2/ERBB2) [55]. The traditional framework for clinical breast cancer classification encompasses three major types: (1) luminal ER-positive and PR-positive (additionally subdivided into luminal types A and B based on histochemical staining for the proliferation marker protein Ki-67 (MKI67) or genetic profiling); (2) HER2-positive; (3) triple-negative breast cancer (TNBC) [56]. Nevertheless, the appreciation of the tumor-immune dynamic relationship, breakthroughs in omics technologies and next-generation sequencing, and inclusion of predictive and prognostic genomic and immunomarkers have enabled better comprehension of the malignant ecosystem and progression pathways, refined diagnostic and patient-stratification algorithms, and most importantly, revolutionized the therapeutic landscape, thus providing novel opportunities for breast cancer management [57].

Etiopathology of breast cancer is rather complex. A variety of intricately interrelated modifiable and non-modifiable factors, including genetics, environmental exposures, and physiological, sociobiological and lifestyle determinants, may increase susceptibility or encourage disease development [58]. Female sex is one of the major risk factors, predominantly due to elevated hormonal stimulation and increased vulnerability of breast tissue to estrogen-progesterone balance disruption. Male breast cancer accounts for approximately 1% of all cases, and the most common being the ER-positive type [59]. Currently, nearly 80% of breast cancer patients are older than 50, while more than 40% of affected individuals are older than 65 years. Epidemiological data reveal associations between age at diagnosis and certain cancer molecular subtypes. Aggressive, recurrence-prone, resistant TNBC is most frequently detected in populations less than 40 years of age, whereas among patients above 70 years the luminal A type is most frequently detected [60]. A significantly higher incidence rate of breast cancer is observed among individuals with a positive family history, with 13-19% of patients having a first-degree relative affected with the same or related malignancy [61]. Familial clustering of breast cancer was estimated to be approximately 73% attributed to heritable components and 27% to environmental triggers. Most inherited cases due to predisposing genetic mutations are associated with two autosomal dominant high-risk genes: BRCA1 (BReast CAncer gene 1) and BRCA2 (BReast CAncer gene 2), located on chromosomes 17(17q21.31) and 13(13q13.1), respectively. Several pathogenic variants in highly penetrant (e.g., TP53, CDH1, PTEN and STK11) and moderately penetrant susceptibility genes (e.g., CHECK2, PALB2, ATM and BRIP1) were also reported to be involved in breast carcinogenesis [62]. Although large-scale genotyping analysis within genome-wide association studies (GWAS) provided significant advancement in this area, a considerable proportion remains unclear. Further research is needed to elucidate the intricate breast cancer genetic background. Extensive scientific evidence corroborates the relationship between an individual’s reproductive history and the risk of breast cancer. Timing, duration and accompanying hormonal fluctuations of particular entities, such as menstruation, pregnancy and breastfeeding may have a considerable impact on the potential induction of oncogenesis in the breast microenvironment. Early menarches, advanced age of full-term pregnancy, absence or short-period breastfeeding, nulliparas or low parity, and late menopause (with a high total lifetime count of menstrual cycles) elevate the breast cancer risk. Additionally, certain breast properties such as high tissue density, personal history of cancerous and non-cancerous lesions, as well as previous chest exposure to radiation were correlated with increased risk of breast cancer occurrence. Other environmental and lifestyle risk factors include smoking, a high-fat diet, excessive alcohol consumption, hormonal replacement therapy, oral contraceptives, obesity and low physical activity [63,64].

### 3.2. Breast Cancer Chemoprevention and Dietary Isoflavone Intake

Accumulating evidence and enhanced comprehension of intricate mechanisms involved in cancerogenesis have directed scientific attention toward the immense potential of chemopreventive strategies and, in particular, compounds that may suppress, arrest or reverse various molecular pathways associated with cancer development and progression. Chemoprevention encompasses a myriad of molecular targets related to distinct stages of the carcinogenic process (i.e., initiation, promotion, and progression) and may be broadly categorized into three major types: primary, secondary and tertiary [65,66]. Primary prevention refers to the exposure of healthy individuals or populations exerting particular risk features but without the overt disease to certain natural or synthetic agents in order to prevent cancer occurrence. Secondary prevention pertains to premalignant lesions and the administration of selected agents to perturb the transition to invasive cancer. Tertiary prevention applies to patients who successfully underwent cancer treatment in order to prevent recurrence or the development of a new primary tumor [67]. Compounds that impede the initial neoplastic transformation are termed as “blocking agents”, whereas those that counteract the progression of benign lesions to advanced-stage cancers are denominated as the “suppressing agents”. Mechanistically, these agents effectuate their chemopreventive properties by a wide spectrum of functions, including the inhibition of carcinogen uptake, induction of cellular detoxifying enzymes and genomic repair pathways, antioxidant activity and free radical scavenging, modulation of epigenetic alterations, downregulation of chronic inflammation, as well as interaction with cellular signaling networks responsible for maintaining the delicate balance between cell survival, differentiation, proliferation, senescence, and death [68].

A multitude of epidemiological studies has associated isoflavones and their dietary sources with various health benefits, including protection against hormone-dependent oncopathologies (such as breast and prostate cancer), cardiovascular disease, disturbances in glycoregulation, osteoporosis, support to weight management/obesity reduction and attenuation of physical and emotional menopausal symptoms [8,69]. Available dietary surveys indicate a great level of variability regarding the intake of total phytoestrogens, particular subclasses of these plant-derived secondary metabolites, and their food sources with regard to geographical and sociocultural determinants. Consumption of foods containing high levels of isoflavones, such as soybeans and soy-based products and other legume seeds (beans, peas, lentils, clover), is commonly associated with traditional Asian dietary patterns rather than with nutritional preferences of the Western world. Despite the considerable inter-study variations, a substantially higher average daily intake of isoflavones per capita is reported for South and East Asian countries (i.e., 20–50 mg/day) than for European countries (0.37–4.5 mg/day) and the USA (0.73–3.3 mg/day) [70,71,72,73]. Interestingly, however, in the last couple of decades, noteworthy changes in isoflavone-related dietary habits were observed: simultaneously with the decreased consumption of soy in favor of animal-sourced protein in Asia due to the adoption of Westernized nutrition, the intake of soy is growing among health-conscious individuals in the USA and Europe. Based on dietary analyses, vegans and vegetarians are a Western-world subgroup with the highest isoflavone intake of approximately 7–12 mg/day [74]. Dominant isoflavone sources in traditional Asian diet are fermented and non-fermented soybeans and first-generation soy products, including tofu, tempeh, miso, soy milk, natto and cheonggukjang. In contrast, soy-based meat and dairy substitutes, as well as various food derivatives with soy flour or soy proteins added as fillers or extenders, prevail in modern Western habitual nutrition [8,75].

Given the biological properties of isoflavones and a striking concomitant geographical discrepancy in isoflavone intake and breast cancer prevalence, researchers have hypothesized the potential role of these compounds in the prevention of mammary tumorigenesis among susceptible populations and among women with or without previous history. In addition to epidemiological-level research, numerous studies explored the benefits of isoflavones in cell cultures, animal and human breast cancer scientific models trying to: (1) elucidate mechanisms of action and tissue-specific dose-dependent response; (2) validate relevant biomarkers; (3) determine efficacy, safety, opportunities, and challenges of controlled preventive and curative interventions. Meta-analyses indicated a positive association between the consumption of high levels of isoflavones and a reduced breast cancer risk in Asian populations, but failed to detect a significant correlation in Western populations, presumably due to low isoflavone intake levels [76,77,78,79]. Nevertheless, in these meta-analyses, there was considerable between-study heterogeneity that may have compromised the reported observations. Key limitations comprise the case-control design in the majority of included studies, inherent drawbacks of the employed exposure evaluation methods, varied range definitions and intake cut-off values, as well as residual confounding related to unreported yet relevant factors. Contrary to other reports, the China Kadoorie Biobank (CKB) study, which enrolled more than 300,000 women residing in 10 diverse regions in China, found no association between soy intake and breast cancer incidence overall. However, based on the further dose–response meta-analysis integrating the CKB study with other prospective studies, each 10 mg/day of soy isoflavone led to a 3% risk reduction [80]. Literature reports accounting for menopausal status and hence endogenous estrogen environment are also conflicting. Although a significant inverse association between the isoflavone intake and breast cancer risk in both pre- and post-menopausal Asian women was reported [78,79], the menopausal status may be an important effect modifier with the protective effect of soy isoflavone intake existing among postmenopausal subjects only [76,81]. Recent meta-analyses summarizing findings from eight prospective studies from 2003 to 2021 concluded that elevated soy consumption and, consequently, isoflavone intake are associated with better breast cancer prognosis and lower occurrence risk regardless of menopause status [82]. According to the systematic review and meta-analysis by combining data derived from 12 scientific articles and 37,275 cases of women with breast cancer, pre-diagnosis soy and isoflavone intake were favorably correlated with the overall survival and reduced recurrence risk among post-menopausal patients exclusively [83]. A number of epidemiological studies evaluated the potential association between isoflavone intake and breast cancer risk stratified by hormone receptor (ER and PR) and HER2 status in the general population. The Shanghai Breast Cancer Study reported a superior risk reduction for ER+/PR+ tumors than for other subtypes [84]. However, in several case-control and cohort studies the protective effect of soy products against breast cancer remained comparable across all ER/PR status subtypes. Concerning the HER2 status, a Japanese case–control study [85] found that elevated intake of soy-based products significantly reduced the risk of HER2-negative breast cancer by 21%, whereas no significant differences with respect to HER2 status were found in another study [86]. A recent study examined the association between the isoflavone intake and breast cancer risk by molecular subtype in 1,709 Korean females featuring a high risk of hereditary breast cancer (i.e., BRCA1/2 mutation carriers and non-carriers with family history and early-onset breast cancer). The study indicated that the high isoflavone intake in this population might act as a preventive factor, particularly in BRCA2-mutated luminal A type and BRCA1-mutated triple-negative breast cancer [87]. A summary of discussed studies is provided in Table 1.

Despite the extraordinary scientific interest, evidence on the association between isoflavone dietary intake and breast cancer incidence, recurrence and survival remains rather inconclusive. Additional investigation is needed to substantiate and further elucidate the postulated isoflavone health benefits. The inconsistency in the reported results on both single-study and meta-analysis levels may be attributed to a number of factors: (1) methodological limitations; (2) variability in experimental design (retrospective vs. prospective); (3) sample size and population characteristics; (4) differences in overall exposure level and timing (childhood/adolescence vs. adulthood); (5) environmental and sociocultural circumstances; (6) the lack of information on the nature, type, source and bioavailability of isoflavones. In general, although promising, findings yielded in observational studies warrant confirmation and further clarification in future research with enhanced data standardization and documentation, reliable sensitivity analysis, larger sample size, and prolonged follow-up duration, precise dietary intake assessment, and carefully controlled confounding factors.

### 3.3. Isoflavone Mechanisms of Action and Impact on Breast Cancer

Isoflavones can mediate the majority of their biological effects via estrogen receptor (ER) signaling pathways due to a significant structural resemblance with the mammalian 17-β-estradiol (E2). Although the importance of estrogens and their receptors in breast cancerogenesis and subsequent tumor development is well-established, the implication of various environmental and synthetic receptor modulators (i.e., phytoestrogens and xenoestrogens) is still a controversial subject and a matter of debate. Isoflavones may interact with both alpha (ERα) and beta receptor (ERβ) isoforms but exert markedly stronger affinity towards the ERβ. Even though the ligand binding cavities of ERα and ERβ differ by only two amino acid positions (replacement of Leu-384 and Met-421 in ERα with Met-336 and Ile-373, respectively, in ERβ), the sequence diversity beyond the pocket residues confers the ERβ to the topology more suitable for the isoflavones [10]. 

In general, the ER binding potency of isoflavones ranges from 10^−4^ to 10^−3^ compared to the primary natural ligand, i.e., estradiol (E2). Appropriate orientation, hydrophobic core and planar chemical structure featuring *p*-hydroxy-substituted aromatic ring approximately 12 Å distant from the 2^nd^ planar hydroxyl group enable isoflavones to mimic endogenous estrogen interaction with the hormone binding pockets of ERs. Specific structural elements shaping spatial, electronic, and topological characteristics of particular isoflavone compounds determine their potency as phytoestrogenic ligands and convey their qualitative and functional properties [88]. Quantitative structure-activity relationship (QSAR) and comparative molecular field research highlighted pharmacophore features significant for receptor binding and activation, including partial charges on atoms C2′, C4′ (ring B) and C7 (ring A), conformational rigidity determinants and molecular orientation [89]. Given that isoflavones may display both estrogenic and anti-estrogenic effects, they are sometimes regarded as natural selective ER modulators (SERMs). The ultimate actions are predisposed by a multitude of factors, including ambient estrogen concentrations and environmental hormonal milieu, relative levels of ERα and ERβ, the local concentration of the studied isoflavone compound(s), the presence of other phyto/xenoestrogens as well as the interference of present co-activators and co-repressors [90]. Nevertheless, the findings of various studies are rather inconsistent, and comprehensive characterization of the intricate bioactivity of isoflavones warrants further research. These incoherencies may be at least partly attributed to substantial qualitative and quantitative variability of isoflavones content and composition in food sources, the complexity of their metabolic transformations as well as inter and intra-individual genetic and physiological differences. Several preclinical rodent tumor models were applied in animal studies exploring the isoflavone influence on breast cancer. Experimental induction of breast cancer may be obtained through genetic engineering leading to spontaneous cancer development by administration of a chemical carcinogen (most commonly 1-methyl-1-nitrosourea (MNU) for protocols aiming to investigate more aggressive mammary oncopathologies or 7,12-dimethylbenz[a]anthracene (DMBA) or by exposure to excess estrogen levels [6]. The inoculation model refers to the transplantation of tumor cells or explants into susceptible murine hosts. The xenograft model is generated in immunodeficient hosts with immortalized human cancer cell lines (commonly MCF-7 or MDA-MB-231) or tumor explants of patient origin, whereas allograft models are created when allogenic cells/tissues are transplanted into immunocompetent syngeneic mice [91]. Each of these models is of great scientific significance, albeit their inherent imperfections and species-specific peculiarities limit the translational relevance of findings to humans. The deficiency of target-site exposure data undoubtedly remains the critical knowledge gap to be addressed in future studies. These data would contribute to better interpretation of *in vitro* results, translation of such findings into *in vivo* relevant context, and eventually their integration with human observational and interventional research in comprehensive risk-assessment analyses.

**Figure 3:**
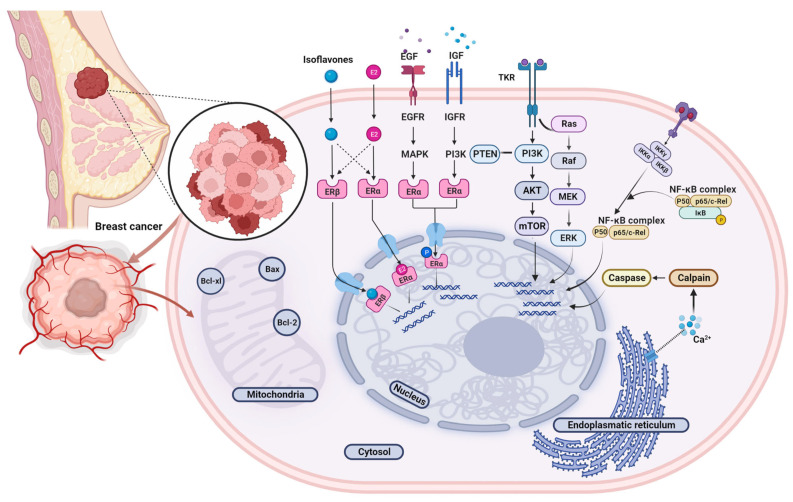
Graphical overview of the isoflavones’ possible molecular targets and cellular signaling pathways in the breast cancer model. Abbreviations: AKT, Protein kinase-B; Bcl-2, B-cell lymphoma 2; Bcl-xL, B-cell lymphoma-extra large; Bax, Bcl-2-associated X-protein; E2, Estradiol; EGF, epidermal growth factor; EGFR, Epidermal growth factor receptor; ERα, Estrogen receptor alpha; ERβ, Estrogen receptor beta; ERK, Extracellular-signal-regulated kinase; IGF, insulin-like growth factor; IGFR, insulin-like growth factor receptor; IKKα, IKKβ and IKKγ, IκB ki-nases; MAPK, Mitogen-activated protein kinases; MEK, Mitogen-activated protein kinase kinase; mTOR, mammalian target of rapamycin; NF-κB, nuclear factor-κB; PI3K, Phosphoinositide 3-kinase; PTEN, Phosphatase and tensin homolog; Raf, Rapidly accelerated fibrosarcoma; Ras, Rat sarcoma, TKR; Tyrosine kinase receptor.

Although the interaction with ER is the most commonly studied isoflavone mechanism of action, there is a growing body of evidence indicating that additional pathways and alternative cascades may also be affected. In addition to ER-mediated signaling, isoflavones may modulate the local hormone levels in breasts and ovaries in a tissue-specific and concentration-dependent manner by affecting the activity or expression of steroidogenic enzymes implicated in estrogen synthesis and metabolism (e.g., cytochrome P450—aromatase (CYP19A1), 3β-hydroxysteroid dehydrogenase and 17β-hydroxysteroid dehydrogenase, cytochrome P450 1A1 and 1B1 and sulfotransferase, thereby altering the conversion of androgenic precursors to estrogens and the dehydrogenation of estrone to estradiol [92]. Furthermore, isoflavones deliver antitumorigenic effects through several ER-independent activities, including apoptosis induction, inhibition of cell proliferation and angiogenesis, support for antioxidant defense and DNA repair mechanisms, inflammation downregulation, and interference in other signal-transduction systems (Figure 3) [75].

Studies indicated dose-sensitive dual (i.e., estrogenic and anti-estrogenic) effects of isoflavones via multimodal mechanistic pathways. Contrasting effects (cell proliferation and apoptosis) on breast cancer cells were detected both *in vitro* and *in vivo*. Due to isoflavone pleiotropic properties, exceptional heterogeneity of breast cancer molecular features and a multitude of modulating factors it is not always clear under what circumstances these plant-derived compounds act favorably against tumors and when they exert adverse effects by promoting cancer cell proliferation [91]. At low doses (≤10 µmol/L), major soy isoflavone genistein displayed estrogen-like effects and was found to stimulate the proliferation of hormone-sensitive breast cancer cell lines and tumors in mouse models [93,94,95]. The observed pro-tumorigenic effect is likely mediated by the activation of the ERα pathway and attributed to genistein acting as a weak estrogen. However, at higher doses genistein was reported to antagonize the progression of breast neoplasms by triggering apoptosis, inducing cell cycle arrest, and impeding angiogenesis employing estrogen-independent pathways, including the activation of caspase and several endoplasmic reticulum stress-regulators, disabling of VEGF signaling, downregulation of matrix metalloproteinases, inhibition of the MEK5/ERK5/NF-kB, protein tyrosine kinase (PTK), mitogen-activated protein kinases (MAPK) and epigenetic modification [96,97,98,99,100].

## 4. Prostate Cancer and Isoflavones

### 4.1. Prostate Cancer—Epidemiology and Risk Factors

Androgens are essential for the regulation of cell survival, development, differentiation, and proliferation within the prostate gland. However, the development of both normal and abnormal prostatic glandular structures is determined by the complex reciprocal stroma-epithelial interactions at endocrine and paracrine levels.

In benign conditions called benign prostatic hyperplasia (BPH), the intensified proliferation of fibroblasts is stimulated by the increased local conversion of testosterone (T) to estradiol (E2) in the stroma. The enlarged cell population of fibroblasts produces larger amounts of dihydrotestosterone (DHT) and tissue growth factors (GF), which activate the proliferation of the epithelium. In this way, a vicious cycle is created, followed by progressive growth of total prostate volume (TPV) and increased prostate-specific antigen (PSA) synthesis [101,102]. A better comprehension of the steroid pathways in prostatic disorders, with an emphasis on intraprostatic androgen levels in particular, may contribute to the personalization and optimization of treatment algorithms and presumably new therapeutic targets. In prostate cancer, stroma-epithelium interaction is maintained. However, multiple mutations and defects alter the cellular signaling pathways, androgen and estrogen receptors, as well as hormonal levels in the tissue.

The vast majority of prostate malignant neoplasms have epithelial origin and differentiation and are considered carcinomas based on histopathological and immunophenotypic attributes [103]. The oncogenesis and pathobiology of prostate cancer are associated with multifarious interactions between inherent germline lineage susceptibility, acquired somatic mutations, and both micro and macro environmental factors. Localized prostate cancer often comprises multiple foci featuring specific genetic alterations with diverse capacities for metastatic colonization and treatment resistance. The identity of cancer-initiating cells in prostatic adenocarcinomas remains controversial, but it is generally acknowledged that during tumorigenesis the epithelium undergoes a series of phenotypic modifications, including cell signaling perturbations, which facilitates the transformation from benign to malignant disease [104]. The most common type of prostate cancer is adenocarcinoma of the glandular acini of the prostate. Given that the largest percentage of glandular acini are located in the peripheral zone of the prostate, prostate cancer most often occurs in the peripheral zone and, in the initial stages, rarely causes urethral obstruction and dysuric disorders [105]. During growth, cancer occupies a larger part of the prostate and, if not detected and treated in time, spreads beyond the prostate borders and metastasizes to the pelvic lymph glands and bones.

Prostate cancer risk substantially increases with aging, and it is predominantly diagnosed in men over the age of 65 and very rarely in those younger than 50 [106,107]. Consequently, prostate cancer incidence is exceptionally high in regions with long life expectancies. Prostate cancer is the most common cancer in men in Europe, America, Australia and Sub-Saharan Africa, accounting globally for approximately 7% of newly diagnosed cancer cases in the male population (15% in developed regions). Additionally, worldwide estimation of prostate cancer-related deaths exceeds 375,000 annually, qualifying it as the fifth leading cause of cancer-associated mortality in men. The general burden of prostate cancer is supposed to increase due to the overall population aging trends and economic growth. Based on the GLOBOCAN 2020 Report [46] estimated region-specific age-standardized incidence rates range from 6.3 to 83.4/100,000 men. The highest incidence levels (exceeding 59.0/100,000 males) are reported for Northern and Western Europe, the Caribbean, Australia/New Zealand, Northern America and Southern Africa, while the lowest rates are found in Asia and Northern Africa. Nevertheless, geographic variability of mortality estimates deviates from the incidence patterns with the highest rates being noted for the Caribbean, Africa and Oceania. Remarkable international variation of prostate cancer epidemiological statistics and temporal trends may be attributed to both genetic and environmental influences, including socioeconomic status, lifestyle and nutrition factors, screening programs and healthcare disparities.

Extensive research endeavors are being conducted with the aim to determine and describe variables that affect the risk of prostate cancer. Identified or postulated risk factors for the development of this malignancy are numerous and heterogeneous. Currently acknowledged determinants that modulate the susceptibility to prostate carcinogenesis include genetic, androgen-associated, infectious, inflammatory, ethnic, lifestyle and nutrition pathways [108,109,110]. A better comprehension of modifiable risk factors may focus and guide the creation and implementation of preventative measures and targeted interventions on the public health and individual level.

Epidemiologic studies indicated a familial clustering of prostate cancer, which proposed that risk is significantly increased among men with an affected first-degree relative. The prostate cancer relative risk increases with the increasing number of family members with prostate cancer in medical history, the grade of genetic relatedness of affected individuals, and the decreasing age at diagnosis [111,112]. Both common environmental exposures and genetic influences may contribute to the familial aggregation of prostate cancer. Higher risk is determined in individuals with an affected brother in comparison with those having an affected father [113]. These generational discrepancies may be attributed to alterations in environmental, nutritional and occupational exposures over the course of time, especially during childhood and adolescence, and the contribution of maternal genetic inheritance.

Based on paleopathologistic research, Homo sapiens was an obligatory herbivore for over 300,000 years of existence until the last 12,000–15,000 years, when it began to introduce the meat of domestic animals into the habitual diet, concomitantly reducing the intake of plant foods. This significant change in evolutionary dietary trajectory is now hypothesized to be an important factor in the rising incidence of breast and prostate cancer in humans. Although discrepancies in national screening practices may impact the detection rate of prostate cancer, the cross-country comparisons of epidemiologic data support the importance of nutrition and dietary pattern as risk or protective factors. The incidence of prostate cancer in Chinese immigrants who live in Western countries is significantly higher compared to Chinese living in China [114,115,116]. Albeit the differences in medical systems, screening programs, and cancer registries cannot be discarded, it is reasonable to postulate that the abandonment of traditional diet and lifestyle may also be significant. Furthermore, a similar trend of increasing incidence of prostate cancer is noted in large Chinese cities, where the intake of meat and animal fats increased by almost 600% in the period from 1960 to 2000 [117].

The association between infections, infection-mediated chronic inflammatory process, and oncogenic transformation is well-acknowledged. Accumulating evidence from basic science, clinical research and epidemiological studies suggests that inflammation may affect prostate carcinogenesis. Although causative agents and drivers of intraprostatic tumor-promoting inflammation remain elusive, it is hypothesized that proliferative inflammation-induced atrophy featuring cell infiltrates elicited by unknown stimuli, which potentially include microbial pathogens, might serve as a precancerous lesion leading to prostatic intraepithelial neoplasia and/or prostate cancer [118].

Copious scientific evidence supports the notion of the vitamin D signaling-cascade role in prostate cancer and other oncopathologies. Epidemiologic data suggest that vitamin D status may be significant in the causation of prostate cancer and a plausible target for the prevention and treatment of this malignancy. A higher risk for prostate cancer and/or poor prognosis was reported for population groups with low vitamin D intake and 25(OH)D_3_ serum concentration, indicating (subclinical) vitamin D deficiency, including elderly, men with limited sun exposure or living in higher latitudes, and African-Americans whose skin pigmentation is associated with decreased intracutaneous vitamin D synthesis [119,120]. Vitamin D receptor is found in both normal and cancerous prostate tissue, and it is postulated that its expression and certain polymorphisms may represent predictive markers of prostate cancer progression and outcome beyond standard clinical indicators [121,122]. The potential translation of these observations into preventive or prostate cancer management strategies warrants further research and carefully designed clinical trials.

The increase in human lifespan is another factor that enables the cumulative effect of carcinogens, thus imposing significant global health challenges. In the Neolithic period, the average life expectancy of humans was approximately 20 years, and nowadays, it is estimated to be 70–80 years [123]. To accurately assess the social burden caused by a particular disease and thereby effectively allocate healthcare resources, it is essential to comprehend the impact of shifting population age structure. The prevalence of prostate cancer undeniably and substantially increases with age, and based on autopsy studies, the estimated rate varies from 5% among men under 30 years of age to almost 59% among men older than 79 years [124]. Cancer statistics and population projections indicate that prostate cancer will probably become more prevalent in a globally aging population. Nevertheless, aging-adjusted morbidity rates in the Asian region and the developing Western countries are anticipated to rise considerably more than in the developed Western world, where they have already plateaued and may even be declining with time.

Several studies demonstrated an association between obesity and an increased risk of developing prostate cancer. Furthermore, obesity may affect tumor growth, the prognosis of the disease, recurrence rates and overall mortality estimates [125,126,127,128,129]. Although the association between prostate cancer and the alteration of hormone status mediated by obesity and endocrine activity of adipose tissue is still controversial, it has been shown that obese men exert increased estradiol, insulin, leptin and free IGF-1 levels concomitantly with the decreased free T concentration, and declining luteinizing hormone (LH) pulse amplitude [130]. Moreover, the multifaceted interplay between obesity-induced low-grade systemic inflammation and immune response may be a significant factor in the progression of prostate cancer. It is hypothesized that several chemokines and cytokines secreted from adipose depots may cause alterations of the local immune cellular profile in the tumor microenvironment, including myeloid-derived suppressor cells, cancer-associated neutrophils, macrophages, B-cells, immunoglobulins and the complement system, thereby promoting proliferation, invasion and metastatic seeding. Another possible biological vehicle of obesity-related prostate cancerogenesis refers to host immune-system modulation triggered by intestinal microbiome alterations. Given the scarcity of available data, additional research is needed to fully elucidate precise mechanisms in the background of this phenomenon.

### 4.2. The Effects of Isoflavones on Prostate Cancer

Several investigators reported that genistein might suppress the PSA expression in prostate cancer cell lines and arrest the cell cycle progression, presumably by interfering with tyrosine kinases vital for mitogenic signal transduction [131,132]. A histoculture study of surgical specimens of human BPH and prostate cancer was performed using the pre-established hormone-sensitive three-dimensional collagen gel matrix. It was demonstrated that at a dosage of 1.25–10 μg/mL genistein inhibits the growth of both BPH and prostate cancer tissues in a dose-dependent manner as measured by ^3^H-thymidine DNA incorporation per mg protein, with a negligible additional effect in higher concentrations [133].

**Figure 4:**
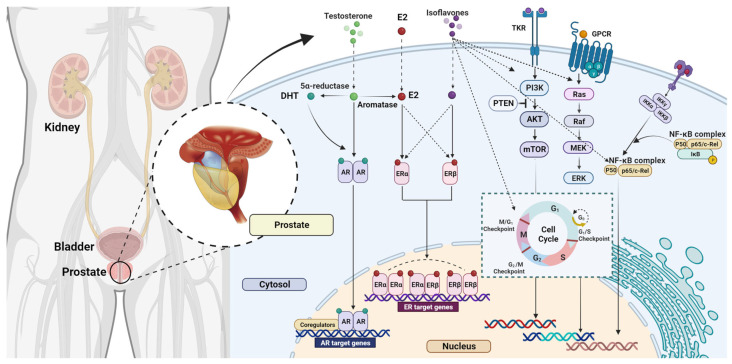
Graphical overview of the isoflavones’ possible molecular targets and cellular signaling pathways in the prostate cancer model. Abbreviations: AKT, Protein kinase-B; AR, Androgen receptor; DHT, Dihydrotestosterone; E2, Estradiol; ERα, Estrogen receptor alpha; ERβ, Estrogen receptor beta; ERK, Extracellular-signal-regulated kinase; GPCR, G protein-coupled receptor; IKKα, IKKβ and IKKγ, IκB kinases; MEK, Mitogen-activated protein kinase kinase; mTOR, mammalian target of rapamycin; NF-κB, nuclear factor-κB; PI3K, Phosphoinositide 3-kinase; PTEN, Phosphatase and tensin homolog; Raf, Rapidly accelerated fibrosarcoma; Ras, Rat sarcoma; TKR, Tyrosine kinase receptor.

The primary action of genistein on prostate cancer cells at pharmacological doses appears to be the induction of apoptosis. Substantial evidence derived from *in vitro* studies suggests that genistein inhibits the growth and metastatic dissemination of diverse types of both hormone-dependent and hormone-independent prostate and breast tumors, displaying the half-maximal inhibitory concentration (IC_50_) ranging from 5 to 40 nM (i.e., 2-10 mg/mL) [131,134,135,136]. The mechanism by which genistein acts as an anticancer agent is probably based on targeting multiple oncogenic pathways, including PLK1, EGFR, Wnt, Akt, JAK/STAT, epithelial-mesenchymal transition, and ER modulation (Figure 4). Furthermore, genistein inhibits the activity of DNA topoisomerase II and increases *in vitro* concentration of TGFb (transforming growth factor b), which can inhibit the growth of malignant cells [137,138].

The growth-inhibitory potential of genistein and vitamin D compounds on prostate epithelial cells was examined. Genistein and the hormonally active form of cholecalciferol synergistically suppressed the proliferation of primary human prostatic epithelial cells (HPEC) and prostate cancer cells, according to the isobolographic study. Based on the flow-cytometry analysis, genistein induced an arrest in the G(2)M phase of the cell cycle, while 1α,25-dihydroxycholecalciferol and low-calcemic 25-hydroxycholecalciferol caused an arrest in the G(1/0) phase [139].

Another study demonstrated that in an androgen-depleted LNCaP cell system, genistein dose-dependently inhibits the DHT-induced expression of the prostate androgen-regulated transcript 1 gene (*PART-1*) with complete inhibition obtained at the concentration of 50 mM. These results suggested that *PART-1* may serve as a promising marker for assessing the effect of isoflavones (and soy products) in prostate cancer prevention. Nevertheless, additional research efforts, in particular animal studies, are required to explore whether these compounds inhibit prostate tumor growth via *PART-1* expression inhibition *in vivo* [140]. Studies indicated that genistein inhibits tumor growth and induces apoptosis at concentrations ≤20 mM in both androgen-dependent and independent human prostate adenocarcinoma cell lines, such as LNCaP, DU-145 and PC3 [141,142,143]. Moreover, *in vitro* research suggested that genistein acts by inhibiting NF-κB (nuclear factor kappa-light-chain-enhancer of activated B cells) in various cells, suppresses metalloproteinases associated with cancer and inhibits telomerase activity via transcriptional downregulation of human telomerase reverse transcriptase (hTERT) [144,145].

Given that chemopreventive compounds should be administered at biologically effective doses over extended periods of time without causing toxicity and health-compromising adverse effects, research on genistein considering mechanisms of action at physiologically relevant concentrations is of particular importance. It was found that physiological concentrations of genistein (comparable to those observed in the sera of Asian male habitual soy consumers) decrease the androgen receptor (AR) expression on mRNA and protein levels in the androgen-sensitive LNCaP line, coupled with a dose-dependent reduction of PSA secretion. Pure anti-estrogen caused the abrogation of the inhibitory effect of genistein, confirming the hypothesis that the downregulation of AR was mediated by the ERβ [146]. Tumor-suppressive features of genistein on PC3 and DU145 cells were investigated by conducting a functional assay cluster analysis. Genistein was reported to inhibit growth of prostate cancer cells by downregulating the HOX transcript antisense RNA (HOTAIR) gene located on chromosome 12. Based on luciferase reporter assay, real-time PCR and long non-coding RNA profiling, HOTAIR was directly targeted by miR-34a, significantly regulated by genistein, and compared to normal prostate cells its expression was elevated in castration-resistant cancer cell lines. Knockdown of HOTAIR induced apoptotic cascade, diminished prostate cancer cell proliferation, migration potential and invasiveness. Additionally, bioinformatic analysis and mRNA array data implied that MMP9 and VEGF genes, key components of the KEGG pathway, are among genistein’s targets [147]. Genistein also modulates the expression of several microRNAs (miRNAs), thus contributing to a better comprehension of its pleiotropic biological effects and antitumorigenic potential. Genistein may up-regulate the expression of the tumor suppressor miR-574-3p in clinical prostate cancer samples and prostate cancer cell lines, thereby causing apoptosis through Bcl-xL, caspase-9 and caspase-3 pathways regulation [148].

The effects of isoflavonoids and other phytoestrogens on castration-resistant prostate cancer were discussed in a review article [149]. Androgen ablation represents the gold-standard therapy for the advanced non-organ-confined illness with survival-prolonging potency but lacking a curative perspective. The AR-positive human prostate cancer cell line LNCaP (derived from a metastatic prostate cancer lymph node lesion featuring androgen-sensitive growth and a mutation in the ligand binding domain of the AR) is the principal culture model used for investigating the complex interaction of ER and AR axis in prostate cancer. The gain-of-function T877A mutation expands the range of activating ligands for the AR and assigns a specific key castration-resistant prostate cancer pathway in these cells. Functional studies of isoflavones on this representative model indicated that these compounds exert ER ligand capacity with a preference for the ERβ subtype leading to tumor-suppressing activity with an anti-androgenic signature. Apparently, competitive binding to the AR is not how phytoestrogens mediate their effects. The ERβ is virtually silenced during prostate carcinogenesis and phytoestrogen treatment causes its up-regulation. ER-knock-down essentially mimics the androgen stimulation, and in an intricate hormone receptors’ cross-talk, the re-expression of the ERβ results in lower expression of the following: recognized androgenic markers PSA and PCA3, the androgen co-activator PDEF and the insulin-like growth factor-1 receptor (IGF-1). Given that ERβ release from epigenetic silencing elicits beneficial antiandrogenic effects in prostate cancer models, the subtype-selective ER ligands of both natural and synthetic origin may have a significant role in novel preventive and curative strategies for this oncopathology [150,151].

Critically analyzing available literature sources and acknowledging their chemical features, dietary sources, bioavailability and safety profile, Spagnuolo et al. provided a summarized overview of the genistein chemopreventive molecular mechanisms of action [152]. Copious evidence supports the notion of its multifaceted effects on apoptosis induction, cell cycle arrest, and inhibition of angiogenesis and metastasis. Target signaling pathways include caspases, Bax (B cell lymphoma 2 (Bcl-2)-associated X protein), KIF20A (Bcl-2, kinesin-like protein 20A), ERK1/2 (extracellular signal-regulated kinase 1/2), NF-κB (nuclear transcription factor κB), MAPK (mitogen-activated protein kinase), IκB (inhibitor of NF-κB), Wnt/β-catenin (Wingless and integration 1 β-catenin) and PI3K/Akt (phosphoinositide 3 kinase/Akt) (Figure 4). Furthermore, genistein may act synergistically with some well-known chemotherapeutics, such as adriamycin, docetaxel, and tamoxifen and potentiate their efficacy without causing adverse effects. Despite evident benefits, genistein has two limiting characteristics—high pleiotropy coupled with relatively low bioavailability. Therefore, there is a need for further research in order to clarify genistein’s pharmacodynamics and pharmacokinetics, elucidate supplementary molecular targets, define effective cancer-specific therapeutic dosage, explore approaches to increase its bioavailability and evaluate possible interactions with other pharmacological entities [152].

#### 4.2.1. Animal Studies

A dose of 1 mg genistein/g food was reported to significantly reduce prostate weight in rats and downregulate the EGF pathway and ErbB2/Neu receptor without toxic/adverse effects for the host in a 3-week treatment [153]. Genistein was suggested to inhibit cancer cell growth by modulating transforming growth factor (TGF)β-1 [154]. Another mechanism by which genistein counteracts prostate growth in rats is the inhibition of increased 5αR activity induced by exposure to a high-fat diet [155].

The transgenic adenocarcinoma mouse prostate (TRAMP) model was created as a crucial research tool for better comprehension of the development of adenocarcinomas. New opportunities for chemoprevention studies and the development of methods to counteract particular genetic determinants of cancer susceptibility were facilitated by these genetically modified animals. In situ and invasive prostate cancer are both developed in TRAMP mice, mimicking the comprehensive gamut of human prostate cancer progression from prostatic intraepithelial neoplasia to massive multinodular malignancy, as well as androgen-independent illness. Studies conducted on these autochthonous prostate cancer rodent systems proved that isoflavone-abundant diet leads to a significant decrease in tumor growth, incidence of spontaneous tumor formation and the occurrence of prostate cancer metastases [156,157,158]. Studies showed that high concentrations of genistein administered subcutaneously in prostate cancer-implanted animals might exert inhibitory action towards a number of proteins involved in primary tumor growth and apoptosis, which was not seen at doses relevant to the human diet [159]. Given that mortality from prostate cancer is attributable to advanced metastatic disease, regulation of the metastatic cascade (i.e., the sequence of steps malignant cells must undergo to escape the primary organ, disseminate, implant, survive and divide in a distant location, thus forming a secondary mass) is of paramount importance. According to several *in vitro* discoveries, corroborated by both *in vivo* animal research and early-phase human clinical trials, genistein can modulate metastatic potential markers and reduce human cancer metastasis. At lower concentrations comparable to those obtained through dietary consumption, genistein may suppress the prometastatic processes of cancer cellular detachment, adhesion, migration, invasion, and finally, implantation and growth at the distant site through a number of intricately interrelated pathways [160,161].

A study investigating the impact of genistein on sex steroid receptor expression in rats demonstrated that both short-term and life-long exposure to amounts that approximate physiological concentrations in humans consuming a soy-rich diet cause a significant reduction of AR, ER-α and ER-β mRNAs in the dorsolateral prostate, without evident histomorphological and functional toxicity to the male reproductive system. Evidence of combined down-regulation of sex steroid receptor expression and growth factors signaling cascades imply that genistein may concomitantly modulate endocrine and paracrine tissue growth [162].

Cumulative results derived from seven studies featuring transgenic adenocarcinoma of mouse prostate (TRAMP) mice explored the extent of genistein effects that could be attributed to estrogenic activity. Although the results corroborated the premise of protective ERβ and prooncogenic ERα, dietary genistein reduced the incidence of cancer only in the ER wild-type (WT)/TRAMP mice but not in transgenic mice lacking functional ERα or ERβ, i.e., ER knockout (KO)/TRAMP mice. These findings suggest that genistein may require both receptors to exert protective effects on prostate carcinogenesis [163].

#### 4.2.2. Human Studies

Several studies have addressed the potential hormonal impact of soy consumption among men exploring both beneficial and adverse effects. Monitoring of plasma levels of steroid compounds in men who consumed 1.06 L of soy milk per day for a one-month period showed a decrease in androgen metabolism, manifested as the decline of DHT metabolite 3α,17β-ADG (androstanediol glucuronide) concentration [164]. The absence of clinically significant behavioral or physical evidence of genistein toxicity for doses up to 16 mg/kg body weight was found in a safety study featuring single administration of purified unconjugated isoflavones to healthy men. Despite exceeding manifold dietary-relevant intake levels these concentrations did not cause alteration of the hypothalamic-pituitary-gonadal axis, estrogenic or antiestrogenic symptoms. Pharmacokinetic evaluation of the applied isoflavone mixture confirmed rapid plasma clearance of both genistein and daidzein and predominant urine excretion. The estimated mean elimination half-lives were 3.2 h and 4.2 h for free forms of genistein and daidzein, respectively [165].

The level of isoflavones in prostate tissue was obtained after TURP (transurethral resection of the prostate) and radical cystoprostatectomy using gas chromatography-mass spectrometry (GS-MS). Prostate volumes were ≥40 mL and <25 mL in the TURP group and in the cystoprostatectomy control group, respectively. No difference was found in the concentration of enterodiol, enterolactone, equol and daidzein in BPH tissue and normal prostate tissue. However, a significantly lower concentration of genistein was found in BPH than in the control group [166].

Over a 12-month intervention a soy protein drink was administered to a group of 81 older healthy men with low PSA. In this double-blinded, parallel-arm trial, subjects were randomly assigned to either consume a soy protein drink providing 83 mg of isoflavones in a daily dose or a beverage with negligible isoflavone content. Based on the radioimmunometric assay, no statistically significant differences in PSA levels were recorded after 12 months [167].

In a prospective study conducted in Hong Kong, 176 participants with BPH and lower urinary tract symptoms were randomly allocated to either intervention group (receiving 40 mg of isoflavones per day; Soylife 40) or placebo-control group in a double-blind manner. Patients tolerated isoflavones well over 12 months, but they were only slightly superior to placebo in terms of clinical improvement and laboratory analyses [168].

The striking international and interethnic disparity in the prostate cancer incidence rates, coupled with the increased risk in migrants moving from low to high-risk areas, suggest that modifiable environmental determinants, such as certain dietary agents, might modulate the risk of disease development and progression. A wide variety of molecular signaling pathways are implicated in prostate carcinogenesis and disease progression, and multiple lines of evidence have emerged concerning the anticancer and therapeutic benefits of nutritional factors. Substantial epidemiological data indicate a significantly lower occurrence of prostate cancer in Asia, where soy-based foods are traditionally major diet staples [169,170]. These studies aroused research interest and inspired the hypothesis that soy isoflavones may exert a chemopreventive potential relevant to prostate cancer. The fact that latent prostate cancer is twice as common in the USA as it is in Japan, but the incidence of clinically manifested prostate cancer is up to 15 times higher, supports this hypothesis by indicating that there may be some factors that prevent or delay the onset of clinically significant prostate cancer in Japanese men [116]. Approximately a 100-fold variation was determined in age-adjusted prostate cancer mortality rates between certain geographic and racial groups underpinning the prominence of environmental exposure in the etiology of this oncopathology.

In 1998 a large multinational cross-sectional study exploring predictive variables for prostate cancer mortality found that regular consumption of soy products is significantly protective [171]. Despite encouraging epidemiological and preclinical data, it is challenging to draw definite conclusions regarding the clinical efficacy of isoflavones due to great inter-study variability in terms of research designs, limited samples, insufficient treatment duration and the absence of standardized pharmaceutical formulations [172]. A meta-analysis conducted in 2014 to assess the efficacy and safety of soy isoflavones among men with histologically confirmed prostate cancer or at identified risk for the development of this malignancy indicated that there might be scientific support for epidemiological findings implying the role of soy isoflavones in prostate cancer risk reduction, although most studies included were underpowered and had considerable limitations. Nonetheless, understanding of isoflavone impact on sex hormone endpoints and PSA levels remained unclear [173].

Another meta-analysis showed that high consumption of genistein and daidzein, as well as elevated serum enterolactone concentration, were correlated with a significant prostate cancer risk reduction [174]. A population-based case-control study found phytoestrogen subclass inconsistencies regarding the effect on prostate cancer. Specifically, an inverse association was determined for isoflavones and genistein, whereas a high intake of lignans (particularly lariciresinol, pinoresinol, matairesinol and secoisolariciresinol) was related to an increased prostate cancer risk [175].

A trial designed to investigate the effects of acute exposure to a dietary supplement containing 160 mg of standardized red clover-derived dietary isoflavones in men with clinically significant prostate cancer prior to radical prostatectomy found significantly higher cancer cell apoptosis in the intervention group [176]. In another trial, patients with confirmed prostate neoplasia were treated with multiple-dose soy formulations containing 300/600 mg of genistein and 150/300 mg of daidzein for three months. Oral administration of these soy formulations led to a 32% decrease in serum DHT concentration and relatively mild estrogenic effects [177]. In a prospective multicenter trial, male subjects with localized prostate cancer were randomized to receive either cholecalciferol (200,000 IU in a single dose at the starting point of the study) and genistein (600 mg daily) or placebo during the pre-prostatectomy period. Quantitative immunohistochemistry analyses revealed increased apoptosis and AR expression in the treatment-receiving group compared to the placebo arm. Given that AR signaling plays a critical role in prostate cell growth, differentiation, and function, further research is warranted to elucidate the therapeutic potential and the bioactivity of the applied combination [178].

## 5. Conclusions

A broad spectrum of molecular signaling pathways and oncogenic cascades are implicated in carcinogenesis and disease progression in prostate and breast tissue. Diverse research avenues have explored the isoflavone capacity to prevent the neoplastic transformation or disrupt the biology of malignant cells leading to multiple lines of evidence supporting the notion of their chemopreventive potential. In addition to most extensively studied tissue-specific, concentration-sensitive hormone-mediated activities, substantial evidence emerging from both *in vitro* and *in vivo* studies imply that isoflavones may deliver anticancer effects by several alternative hormone-independent mechanisms, including apoptosis induction, cell-cycle hindrance, inhibition of angiogenesis, inflammation downregulation, antioxidant defense promotion, support for DNA repair mechanisms, and interference with other signal-transduction cascades. Due to isoflavone pleiotropic properties, heterogeneous molecular signature of breast and prostate oncopathologies, and a plethora of additional relevant risk-modulating factors, comprehensive characterization of the intricate mechanistic bioactivity of isoflavones warrants further research.

## Figures and Tables

**Table 1:** Summary of discussed epidemiological studies/meta-analysis on isoflavones intake and breast cancer in women.

Ref.	Ref. Period	Study Type/Design	Main Findings ^1^
[76]	No explicit indication in the search protocol; includes studies published from 1992 to 2010	Meta-analysis (22 studies, 7 cohorts, and 15 case-control studies)	For the high-dose category intake of isoflavones reduced the BCa risk in Asian populations (combined RR/OR = 0.68, 95% CI: 0.52–0.89) but not in Western populations (combined RR/OR = 0.98, 95% CI: 0.8–1.11)
[77]	1966–2006	Meta-analysis (21 studies: 14 case-control and 7 cohort studies)	The estimated pooled RR of BCa for soy food intake was 0.75, 95% CI: 0.59–0.95. Isoflavone intake resulted in a 20% decrease in risk (RR = 0.81, 95% CI: 0.67–0.99)
[78]	Up to January 2013	Meta-analysis (35 studies: 30 on premenopausal women, including 10 cohort/nested case-control and 20 case-control studies; 31 on postmenopausal women, including 12 cohort/nested case-control and 19 case-control studies)	In Asian countries protective effect of isoflavones intake related to BCa was confirmed in both pre- and post-menopausal women (OR = 0.59, 95% CI: 0.48–0.69 and 0.44–0.74 for pre- and post-menopausal women, respectively); Among postmenopausal women living in Western countries soy isoflavone intake had a marginally significant protective effect (OR = 0.92, 95% CI: 0.83~1.00)
[79]	No explicit indication in the search protocol; includes studies published from 1991 to 2007	Meta-analysis (8 studies for high-soy consuming Asians: 1 cohort and 7 case–control studies; 11 studies in low-soy consuming Western populations: 4 cohort/nested case-control and 7 case-control studies)	Among Asian women results implied a dose-dependent, statistically significant association between BCa RR and habitual soy food intake (approximately 16% risk decrease per 10 mg of isoflavones intake/day). No association was found in low-soy consuming Western populations.
[80]	Prospective study: 2004–2014; meta-analysis: up to 2019	Prospective study and a dose-response meta-analysis (9 studies)	No association was found between soy intake and BCa incidence in the prospective study. Meta-analysis of prospective studies estimated that each 10 mg increment in daily soy isoflavones intake was associated with a 3% RR of BCa.
[81]	Up to September 2010	Meta-analysis (18 prospective studies)	The protective effect of soy isoflavones against BCa risk was only observed among studies conducted in Asian populations (RR = 0.76, 95% CI: 0.65–0.86) but not in Western populations (RR = 0.97, 95% CI: 0.87–1.06). Isoflavone intake was also inversely associated with BCa recurrence (RR = 0.84, 95% CI: 0.70–0.99)
[82]	Up to April 2021	Meta-analysis (8 prospective studies)	Isoflavone consumption of >15 mg/day is favored when compared to 0-15 mg/day (OR = 7.01 (95% CI: 6.58–7.47) with reference to BCa risk.
[83]	Up to February 2018	Meta-analysis (12 studies: 10 prospective cohort studies, 1 case–control study, 1 pooled analysis)	Pre-diagnosis isoflavones intake was significantly associated with reduced risk of total mortality and recurrence. RR was predominantly observed among post-menopausal patients.
[84]	August 1996–March 1998	Population-based case-control study	A 30% BCa RR was observed for women in the highest decile group based on isoflavones intake (OR = 0.66, 95% Cl: 0.46–0.95); superior risk reduction was reported for ER+/PR+ tumors than for other subtypes (OR = 0.44, 95% Cl: 0.25–0.78)
[85]	2003–2005	Case-control study	High intake of soybean products was associated with a decreased risk of ER+ (top tertile OR = 5 0.74; 95% CI, 0.58–0.94), HER2- (top tertile OR = 0.78; 95% CI, 0.61–0.99) and ER+/PR+/HER- BCa (top tertile OR = 0.73; 95% CI: 0.54–0.97)
[86]	1996–2000	Population-based prospective cohort study	Soy/isoflavones intake was associated with a significantly decreased risk of ER+/PR+ BCa in postmenopausal women (HR = 0.72; 95% CI: 0.53–0.96) and decreased risk of ER−/PR− BCa in premenopausal women (HR = 0.46; 95% CI: 0.22–0.97). The risk association did not vary by HER2 status.
[87]	2007–2014	Nationwide multicenter cohort study	Isoflavones intake above 15.50 mg/day was inversely associated with luminal A BCa risk in BRCA2 mutation carriers (HR = 0.14, 95% CI: 0.04–0.50) and triple-negative BCa in BRCA1 carriers (HR = 0.09, 95% CI: 0.02–0.40)

^1^ BCa—breast cancer; BRCA1—BReast CAncer gene 1; BRCA2—BReast CAncer gene2; CI—confidence interval; ER—estrogen receptor; ER+—estrogen receptor positive; ER—estrogen receptor negative; HER2—human epidermal growth factor receptor 2; HER2—human epidermal growth factor receptor 2 negative; HR—hazard ratio; OR—odds ratio; PR—progesterone receptor; PR+—progesterone receptor positive; PR—progesterone receptor negative; RR—risk reduction.

## Data Availability

Data sharing is not applicable to this article.

## References

[1] Coffey D.S. (2001). Similarities of prostate and breast cancer: Evolution, diet, and estrogens. Urology.

[2] Teaford M.F., Ungar P.S. (2000). Diet and the evolution of the earliest human ancestors. Proc. Natl. Acad. Sci. USA.

[3] Go V.L.W., Butrum R.R., Wong D.A. (2003). Diet, Nutrition, and cancer prevention: The postgenomic era. J. Nutr..

[4] Pejčić T., Tosti T., Džamić Z., Gašić U., Vuksanović A., Dolićanin Z., Tešić Ž. (2019). The polyphenols as potential agents in prevention and therapy of prostate diseases. Molecules.

[5] Fuentes N., Silveyra P. (2019). Estrogen receptor signaling mechanisms. Adv. Protein Chem. Struct. Biol..

[6] Hüser S., Guth S., Joost H.G., Soukup S.T., Köhrle J., Kreienbrock L., Diel P., Lachenmeier D.W., Eisenbrand G., Vollmer G. (2018). Effects of isoflavones on breast tissue and the thyroid hormone system in humans: A comprehensive safety evaluation. Arch. Toxicol..

[7] Paterni I., Granchi C., Katzenellenbogen J.A., Minutolo F. (2014). Estrogen receptors alpha (ERα) and beta (ERβ): Subtype-selective ligands and clinical potential. Steroids.

[8] Kim I.S. (2021). Current perspectives on the beneficial effects of soybean isoflavones and their metabolites for humans. Antioxidants.

[9] Szeja W., Grynkiewicz G., Rusin A. (2017). Isoflavones, their glycosides and glycoconjugates. Synthesis and biological activity. Curr. Org. Chem..

[10] Leclercq G., Jacquot Y. (2014). Interactions of isoflavones and other plant derived estrogens with estrogen receptors for prevention and treatment of breast cancer—considerations concerning related efficacy and safety. J. Steroid Biochem. Mol. Biol..

[11] Nešović M., Gašić U., Tosti T., Horvacki N., Nedić N., Sredojević M., Blagojević S., Ignjatović L., Tešić Ž. (2021). Distribution of polyphenolic and sugar compounds in different buckwheat plant parts. RSC Adv..

[12] Pantelić M.M., Dabić Zagorac D., Davidović S.M., Todić S.R., Bešlić Z.S., Gašić U.M., Tešić Ž.L., Natić M.M. (2016). Identification and quantification of phenolic compounds in berry skin, pulp, and seeds in 13 grapevine varieties grown in Serbia. Food Chem..

[13] Fotirić Akšić M., Nešović M., Ćirić I., Tešić Ž., Pezo L., Tosti T., Gašić U., Dojčinović B., Lončar B., Meland M. (2022). Chemical fruit profiles of different raspberry cultivars grown in specific Norwegian agroclimatic conditions. Horticulturae.

[14] Fotirić Akšić M., Nešović M., Ćirić I., Tešić Ž., Pezo L., Tosti T., Gašić U., Dojčinović B., Lončar B., Meland M. (2022). Polyphenolics and chemical profiles of domestic Norwegian apple (*Malus* × *domestica* Borkh.) cultivars. Front. Nutr..

[15] Pantelić M., Dabić Zagorac D., Natić M., Gašić U., Jović S., Vujović D., Popović Djordjević J. (2016). Impact of clonal variability on phenolics and radical scavenging activity of grapes and wines: A study on the recently developed Merlot and Cabernet Franc clones (*Vitis vinifera* L.). PLoS ONE.

[16] Tešić Ž.L., Gašić U.M., Milojković-Opsenica D.M., Jayaprakasha K.G., Patil S.B., Gattuso G. (2018). Chapter 3: Polyphenolic profile of the fruits grown in Serbia. In Advances in Plant Phenolics: From Chemistry to Human Health.

[17] Skrt M., Albreht A., Vovk I., Constantin O.E., Râpeanu G., Sežun M., Osojnik Črnivec I.G., Zalar U., Poklar Ulrih N. (2022). Extraction of polyphenols and valorization of fibers from Istrian-grown pomegranate (*Punica granatum* L.). Foods.

[18] Simonovska B., Vovk I., Andrenšek S., Valentová K., Ulrichová J. (2003). Investigation of phenolic acids in yacon (*Smallanthus sonchifolius*) leaves and tubers. J. Chromatogr. A.

[19] Ivanović M., Albreht A., Krajnc P., Vovk I., Islamčević Razboršek M. (2021). Sustainable ultrasound-assisted extraction of valuable phenolics from inflorescences of *Helichrysum arenarium* L. using natural deep eutectic solvents. Ind. Crops Prod..

[20] Orsini F., Vovk I., Glavnik V., Jug U., Corradini D. (2019). HPTLC, HPTLC-MS/MS and HPTLC-DPPH methods for analyses of flavonoids and their antioxidant activity in *Cyclanthera pedata* leaves, fruits and dietary supplement. J. Liq. Chromatogr. Relat. Technol..

[21] Jug U., Glavnik V., Kranjc E., Vovk I. (2018). High-performance thin-layer chromatography and high-performance thin-layer chromatography–mass spectrometry methods for the analysis of phenolic acids. J. Planar Chromatogr..

[22] Guzelmeric E., Vovk I., Yesilada E. (2015). Development and validation of an HPTLC method for apigenin 7-*O*-glucoside in chamomile flowers and its application for fingerprint discrimination of chamomile-like materials. J. Pharm. Biomed. Anal..

[23] Nedić N., Nešović M., Radišić P., Gašić U., Baošić R., Joksimović K., Pezo L., Tešić Ž., Vovk I. (2022). Polyphenolic and chemical profiles of honey from the Tara mountain in Serbia. Front. Nutr..

[24] Bugeja Douglas A., Nešović M., Šikoparija B., Radišić P., Tosti T., Trifković J., Russi L., Attard E., Tešić Ž., Gašić U. (2022). Melissopalynology analysis, determination of physicochemical parameters, sugars and phenolics in Maltese honey collected in different seasons. J. Serbian Chem. Soc..

[25] Nešović M., Gašić U., Tosti T., Horvacki N., Šikoparija B., Nedić N., Blagojević S., Ignjatović L., Tešić Ž. (2020). Polyphenol profile of buckwheat honey, nectar and pollen. R. Soc. Open Sci..

[26] Fraga C.G., Croft K.D., Kennedy D.O., Tomás-Barberán F.A. (2019). The effects of polyphenols and other bioactives on human health. Food Funct..

[27] Halliwell B., Aeshbach R., Loliger J., Aruoma O.I. (1995). The characterization of antioxidants. Food Chem. Toxicol..

[28] Reuter S., Gupta S.C., Chaturvedi M.M., Aggarwal B.B. (2010). Oxidative stress, inflammation, and cancer: How are they linked?. Free Radic. Biol. Med..

[29] Król-Grzymała A., Amarowicz R. (2020). Phenolic compounds of soybean seeds from two European countries and their antioxidant properties. Molecules.

[30] Liu J., Chang S.K.C., Wiesenborn D. (2005). Antioxidant properties of soybean isoflavone extract and tofu in vitro and in vivo. J. Agric. Food Chem..

[31] Choi E.J., Kim G.H. (2014). The antioxidant activity of daidzein metabolites, *O*-desmethylangolensin and equol, in HepG2 cells. Mol. Med. Rep..

[32] Kalaiselvan V., Kalaivani M., Vijayakumar A., Sureshkumar K., Venkateskumar K. (2010). Current knowledge and future direction of research on soy isoflavones as a therapeutic agents. Pharmacogn. Rev..

[33] Rizzo G. (2020). The antioxidant role of soy and soy foods in human health. Antioxidants.

[34] Srivastava R.K., Tang S.-N., Zhu W., Meeker D., Shankar S. (2011). Sulforaphane synergizes with quercetin to inhibit self-renewal capacity of pancreatic cancer stem cells. Front. Biosci. (Elite Ed.).

[35] Izuegbuna O.O. (2022). Polyphenols: Chemoprevention and therapeutic potentials in hematological malignancies. Front. Nutr..

[36] Rudrapal M., Khairnar S.J., Khan J., Dukhyil A.B., Ansari M.A., Alomary M.N., Alshabrmi F.M., Palai S., Deb P.K., Devi R. (2022). Dietary polyphenols and their role in oxidative stress-induced human diseases: Insights into protective effects, antioxidant potentials and mechanism(s) of action. Front. Pharmacol..

[37] Veitch N.C. (2007). Isoflavonoids of the leguminosae. Nat. Prod. Rep..

[38] U.S. Department of Agriculture Agricultural Research Service: 2015. USDA Database for the Isoflavone Content of Selected Foods, Release 2.1; Nutrient Data Laboratory Home Page. https://data.nal.usda.gov/dataset/usda-database-isoflavone-content-selected-foods-release-21-november-2015/resource/1de757af.

[39] Setchell K.D.R., Maynard Brown N., Desai P.B., Zimmer-Nechimias L., Wolfe B., Jakate A.S., Creutzinger V., Heubi J.E. (2003). Bioavailability, disposition, and dose-response effects of soy isoflavones when consumed by healthy women at physiologically typical dietary intakes. J. Nutr..

[40] Yu L., Rios E., Castro L., Liu J., Yan Y., Dixon D. (2021). Genistein: Dual role in women’s health. Nutrients.

[41] Selvakumar P., Badgeley A., Murphy P., Anwar H., Sharma U., Lawrence K., Lakshmikuttyamma A. (2020). Flavonoids and other polyphenols act as epigenetic modifiers in breast cancer. Nutrients.

[42] Frankenfeld C.L. (2017). Cardiometabolic risk and gut microbial phytoestrogen metabolite phenotypes. Mol. Nutr. Food Res..

[43] Jackson R.L., Greiwe J.S., Schwen R.J. (2011). Emerging evidence of the health benefits of S-equol, an estrogen receptor β agonist. Nutr. Rev..

[44] Hsiao Y.H., Ho C.T., Pan M.H. (2020). Bioavailability and health benefits of major isoflavone aglycones and their metabolites. J. Funct. Foods.

[45] Richelle M., Pridmore-Merten S., Bodenstab S., Enslen M., Offord E.A. (2002). Hydrolysis of isoflavone glycosides to aglycones by beta-glycosidase does not alter plasma and urine isoflavone pharmacokinetics in postmenopausal women. J. Nutr..

[46] Sung H., Ferlay J., Siegel R.L., Laversanne M., Soerjomataram I., Jemal A., Bray F. (2021). Global cancer statistics 2020: GLOBOCAN estimates of incidence and mortality worldwide for 36 cancers in 185 countries. CA Cancer J. Clin..

[47] Harbeck N., Penault-Llorca F., Cortes J., Gnant M., Houssami N., Poortmans P., Ruddy K., Tsang J., Cardoso F. (2019). Breast cancer. Nat. Rev. Dis. Prim..

[48] Heer E., Harper A., Escandor N., Sung H., McCormack V., Fidler-Benaoudia M.M. (2020). Global burden and trends in premenopausal and postmenopausal breast cancer: A population-based study. Lancet Glob. Health.

[49] Porter P. (2008). “Westernizing” women’s risk? Breast cancer in lower-income countries. N. Engl. J. Med..

[50] Ilic L., Haidinger G., Simon J., Hackl M., Schernhammer E., Papantoniou K. (2022). Trends in female breast cancer incidence, mortality, and survival in Austria, with focus on age, stage, and birth cohorts (1983–2017). Sci. Rep..

[51] Becker S. (2015). A historic and scientific review of breast cancer: The next global healthcare challenge. Int. J. Gynecol. Obstet..

[52] Rositch A.F., Unger-Saldaña K., DeBoer R.J., Ng’ang’a A., Weiner B.J. (2020). The role of dissemination and implementation science in global breast cancer control programs: Frameworks, methods, and examples. Cancer.

[53] Duggan C., Dvaladze A., Rositch A.F., Ginsburg O., Yip C.H., Horton S., Camacho Rodriguez R., Eniu A., Mutebi M., Bourque J.M. (2020). The Breast Health Global Initiative 2018 Global Summit on Improving Breast Healthcare Through Resource-Stratified Phased Implementation: Methods and overview. Cancer.

[54] Prat A., Perou C.M. (2011). Deconstructing the molecular portraits of breast cancer. Mol. Oncol..

[55] Polyak K. (2011). Heterogeneity in breast cancer. J. Clin. Investig..

[56] Loibl S., Poortmans P., Morrow M., Denkert C., Curigliano G. (2021). Breast cancer. Lancet.

[57] Bianchini G., De Angelis C., Licata L., Gianni L. (2022). Treatment landscape of triple-negative breast cancer—Expanded options, evolving needs. Nat. Rev. Clin. Oncol..

[58] Łukasiewicz S., Czeczelewski M., Forma A., Baj J., Sitarz R., Stanisławek A. (2021). Breast cancer—Epidemiology, risk factors, classification, prognostic markers, and current treatment strategies—An updated review. Cancers.

[59] Giordano S.H. (2018). Breast cancer in men. N. Engl. J. Med..

[60] McGuire A., Brown J.A.L., Malone C., McLaughlin R., Kerin M.J. (2015). Effects of age on the detection and management of breast cancer. Cancers.

[61] Shiyanbola O.O., Arao R.F., Miglioretti D.L., Sprague B.L., Hampton J.M., Stout N.K., Kerlikowske K., Braithwaite D., Buist D.S.M., Egan K.M. (2017). Emerging trends in family history of breast cancer and associated risk. Cancer Epidemiol. Biomarkers Prev..

[62] Wendt C., Margolin S. (2019). Identifying breast cancer susceptibility genes—A review of the genetic background in familial breast cancer. Acta Oncol..

[63] Sun Y.S., Zhao Z., Yang Z.N., Xu F., Lu H.J., Zhu Z.Y., Shi W., Jiang J., Yao P.P., Zhu H.P. (2017). Risk factors and preventions of breast cancer. Int. J. Biol. Sci..

[64] Daly A.A., Rolph R., Cutress R.I., Copson E.R. (2021). A Review of modifiable risk factors in young women for the prevention of breast cancer. Breast Cancer.

[65] Steward W.P., Brown K. (2013). Cancer chemoprevention: A rapidly evolving field. Br. J. Cancer.

[66] George B.P., Chandran R., Abrahamse H. (2021). Role of phytochemicals in cancer chemoprevention: Insights. Antioxidants.

[67] Mohan Shankar G., Swetha M., Keerthana C.K., Rayginia T.P., Anto R.J. (2022). Cancer chemoprevention: A strategic approach using phytochemicals. Front. Pharmacol..

[68] Chen C., Kong A.-N.T. (2005). Dietary cancer-chemopreventive compounds: From signaling and gene expression to pharmacological effects. Trends Pharmacol. Sci..

[69] Pabich M., Materska M. (2019). Biological effect of soy isoflavones in the prevention of civilization diseases. Nutrients.

[70] Finkeldey L., Schmitz E., Ellinger S. (2021). Effect of the intake of isoflavones on risk factors of breast cancer—A systematic review of randomized controlled intervention studies. Nutrients.

[71] Rizzo G., Baroni L. (2018). Soy, soy foods and their role in vegetarian diets. Nutrients.

[72] Zamora-Ros R., Knaze V., Luján-Barroso L., Kuhnle G.G.C., Mulligan A.A., Touillaud M., Slimani N., Romieu I., Powell N., Tumino R. (2012). Dietary intakes and food sources of phytoestrogens in the European Prospective Investigation into Cancer and Nutrition (EPIC) 24-hour dietary recall cohort. Eur. J. Clin. Nutr..

[73] Mulligan A.A., Welch A.A., McTaggart A.A., Bhaniani A., Bingham S.A. (2007). Intakes and sources of soya foods and isoflavones in a UK population cohort study (EPIC-Norfolk). Eur. J. Clin. Nutr..

[74] Mortensen A., Kulling S.E., Schwartz H., Rowland I., Ruefer C.E., Rimbach G., Cassidy A., Magee P., Millar J., Hall W.L. (2009). Analytical and compositional aspects of isoflavones in food and their biological effects. Mol. Nutr. Food Res..

[75] Ziaei S., Halaby R., Vinjamury S., Sommers E. (2017). Dietary isoflavones and breast cancer risk. Medicines.

[76] Xie Q., Chen M.L., Qin Y., Zhang Q.Y., Xu H.X., Zhou Y., Mi M.T., Zhu J.D. (2013). Isoflavone consumption and risk of breast cancer: A dose-response meta-analysis of observational studies. Asia Pac. J. Clin. Nutr..

[77] Qin L.Q., Xu J.Y., Wang P.Y., Hoshi K. (2006). Soyfood intake in the prevention of breast cancer risk in women: A meta-analysis of observational epidemiological studies. J. Nutr. Sci. Vitaminol..

[78] Chen M., Rao Y., Zheng Y., Wei S., Li Y., Guo T., Yin P. (2014). Association between soy isoflavone intake and breast cancer risk for pre- and post-menopausal women: A meta-analysis of epidemiological studies. PLoS ONE.

[79] Wu A.H., Yu M.C., Tseng C.C., Pike M.C. (2008). Epidemiology of soy exposures and breast cancer risk. Br. J. Cancer.

[80] Wei Y., Lv J., Guo Y., Bian Z., Gao M., Du H., Yang L., Chen Y., Zhang X., Wang T. (2020). Soy intake and breast cancer risk: A prospective study of 300,000 Chinese women and a dose–response meta-analysis. Eur. J. Epidemiol..

[81] Dong J.Y., Qin L.Q. (2011). Soy isoflavones consumption and risk of breast cancer incidence or recurrence: A meta-analysis of prospective studies. Breast Cancer Res. Treat..

[82] Boutas I., Kontogeorgi A., Dimitrakakis C., Kalantaridou S.N. (2022). Soy isoflavones and breast cancer risk: A meta-analysis. In Vivo.

[83] Qiu S., Jiang C. (2019). Soy and isoflavones consumption and breast cancer survival and recurrence: A systematic review and meta-analysis. Eur. J. Nutr..

[84] Dai Q., Shu X.O., Jin F., Potter J.D., Kushi L.H., Teas J., Gao Y.T., Zheng W. (2001). Population-based case-control study of soyfood intake and breast cancer risk in Shanghai. Br. J. Cancer.

[85] Suzuki T., Matsuo K., Tsunoda N., Hirose K., Hiraki A., Kawase T., Yamashita T., Iwata H., Tanaka H., Tajima K. (2008). Effect of soybean on breast cancer according to receptor status: A case-control study in Japan. Int. J. Cancer.

[86] Baglia M.L., Zheng W., Li H., Yang G., Gao J., Gao Y.T., Shu X.O. (2016). The association of soy food consumption with the risk of subtype of breast cancers defined by hormone receptor and HER2 status. Int. J. Cancer.

[87] Sim E.J., Ko K.P., Ahn C., Park S.M., Surh Y.J., An S., Kim S.W., Lee M.H., Lee J.W., Lee J.E. (2020). Isoflavone intake on the risk of overall breast cancer and molecular subtypes in women at high risk for hereditary breast cancer. Breast Cancer Res. Treat..

[88] Turner J.V., Agatonovic-Kustrin S., Glass B.D. (2007). Molecular aspects of phytoestrogen selective binding at estrogen receptors. J. Pharm. Sci..

[89] Mukherjee S., Mukherjee A., Saha A. (2005). QSAR modeling on binding affinity of diverse estrogenic flavonoids: Electronic, topological and spatial functions in quantitative approximation. J. Mol. Struct. Theochem..

[90] Stubert J., Gerber B. (2009). Isoflavones—Mechanism of action and impact on breast cancer risk. Breast Care.

[91] Russo M., Russo G.L., Daglia M., Kasi P.D., Ravi S., Nabavi S.F., Nabavi S.M. (2016). Understanding genistein in cancer: The “good” and the “bad” effects: A review. Food Chem..

[92] Van Duursen M.B.M. (2017). Modulation of estrogen synthesis and metabolism by phytoestrogens in vitro and the implications for women’s health. Toxicol. Res. (Camb.).

[93] Liu R., Yu X., Chen X., Zhong H., Liang C., Xu X., Xu W., Cheng Y., Wang W., Yu L. (2019). Individual factors define the overall effects of dietary genistein exposure on breast cancer patients. Nutr. Res..

[94] Hsieh C.-J., Hsu Y.-L., Huang Y.-F., Tsai E.-M. (2018). Molecular mechanisms of anticancer effects of phytoestrogens in breast cancer. Curr. Protein Pept. Sci..

[95] Allred C.D., Allred K.F., Ju Y.H., Virant S.M., Helferich W.G. (2001). Soy diets containing varying amounts of genistein stimulate growth of estrogen-dependent (MCF-7) tumors in a dose-dependent manner. Cancer Res..

[96] Ahn S.Y., Jo M.S., Lee D., Baek S.E., Baek J., Yu J.S., Jo J., Yun H., Kang K.S., Yoo J.E. (2019). Dual effects of isoflavonoids from Pueraria lobata roots on estrogenic activity and anti-proliferation of MCF-7 human breast carcinoma cells. Bioorg. Chem..

[97] Li Z., Li J., Mo B., Hu C., Liu H., Qi H., Wang X., Xu J. (2008). Genistein induces cell apoptosis in MDA-MB-231 breast cancer cells via the mitogen-activated protein kinase pathway. Toxicol. Vitro.

[98] Yu X., Zhu J., Mi M., Chen W., Pan Q., Wei M. (2012). Anti-angiogenic genistein inhibits VEGF-induced endothelial cell activation by decreasing PTK activity and MAPK activation. Med. Oncol..

[99] Zhang X., Cook K.L., Warri A., Cruz I.M., Rosim M., Riskin J., Helferich W., Doerge D., Clarke R., Hilakivi-Clarke L. (2017). Lifetime genistein intake increases the response of mammary tumors to tamoxifen in rats. Clin. Cancer Res..

[100] Li Y., Meeran S.M., Patel S.N., Chen H., Hardy T.M., Tollefsbol T.O. (2013). Epigenetic reactivation of estrogen receptor-α (ERα) by genistein enhances hormonal therapy sensitivity in ERα-negative breast cancer. Mol. Cancer.

[101] Lee K.L., Peehl D.M. (2004). Molecular and cellular pathogenesis of benign prostatic hyperplasia. J. Urol..

[102] Pejčić T., Tosti T., Tešić Ž., Milković B., Dragičević D., Kozomara M., Čekerevac M., Džamić Z. (2017). Testosterone and dihydrotestosterone levels in the transition zone correlate with prostate volume. Prostate.

[103] Sandhu S., Moore C.M., Chiong E., Beltran H., Bristow R.G., Williams S.G. (2021). Prostate cancer. Lancet.

[104] De Marzo A.M., DeWeese T.L., Platz E.A., Meeker A.K., Nakayama M., Epstein J.I., Isaacs W.B., Nelson W.G. (2004). Pathological and molecular mechanisms of prostate carcinogenesis: Implications for diagnosis, detection, prevention, and treatment. J. Cell. Biochem..

[105] Ali A., Du Feu A., Oliveira P., Choudhury A., Bristow R.G., Baena E. (2022). Prostate zones and cancer: Lost in transition?. Nat. Rev. Urol..

[106] Stephenson A., Klein A., Wein A., Kavouss L., Partin A., Peters C. (2016). Epidemiology, etiology, and prevention of prostate cancer. Campbell- Walsh Urology.

[107] Gandaglia G., Leni R., Bray F., Fleshner N., Freedland S.J., Kibel A., Stattin P., Van Poppel H., La Vecchia C. (2021). Epidemiology and prevention of prostate cancer. Eur. Urol. Oncol..

[108] Patel A.R., Klein E.A. (2009). Risk factors for prostate cancer. Nat. Clin. Pract. Urol..

[109] Bostwick D.G., Burke H.B., Djakiew D., Euling S., Ho S.M., Landolph J., Morrison H., Sonawane B., Shifflett T., Waters D.J. (2004). Human prostate cancer risk factors. Cancer.

[110] Gann P.H. (2002). Risk factors for prostate cancer. Rev. Urol..

[111] Carter B.S., Beaty T.H., Steinberg G.D., Childs B., Walsh P.C. (1992). Mendelian inheritance of familial prostate cancer. Proc. Natl. Acad. Sci. USA.

[112] Giri V.N., Beebe-Dimmer J.L. (2016). Familial prostate cancer. Semin. Oncol..

[113] Zeegers M.P.A., Jellema A., Ostrer H. (2003). Empiric risk of prostate carcinoma for relatives of patients with prostate carcinoma: A meta-analysis. Cancer.

[114] Yu H., Harris R.E., Gao Y.T., Gao R., Wynder E.L. (1991). Comparative epidemiology of cancers of the colon, rectum, prostate and breast in Shanghai, China versus the United States. Int. J. Epidemiol..

[115] Kimura T. (2012). East meets West: Ethnic differences in prostate cancer epidemiology between East Asians and Caucasians. Chin. J. Cancer.

[116] Shimizu H., Ross R.K., Bernstein L., Henderson B.E., Mack T.M., Yatani R. (1991). Cancers of the prostate and breast among Japanese and white immigrants in Los Angeles county. Br. J. Cancer.

[117] Zhang J., Dhakal I.B., Zhao Z., Li L. (2012). Trends in mortality from cancers of the breast, colon, prostate, esophagus, and stomach in East Asia: Role of nutrition transition. Eur. J. Cancer Prev..

[118] De Martel C., Ferlay J., Franceschi S., Vignat J., Bray F., Forman D., Plummer M. (2012). Global burden of cancers attributable to infections in 2008: A review and synthetic analysis. Lancet. Oncol..

[119] Giovannucci E. (2005). The epidemiology of vitamin D and cancer incidence and mortality: A review (United States). Cancer Causes Control.

[120] Robles L.A., Harrison S., Tan V.Y., Beynon R., McAleenan A., Higgins J.P., Martin R.M., Lewis S.J. (2022). Does testosterone mediate the relationship between vitamin D and prostate cancer progression? A systematic review and meta-analysis. Cancer Causes Control.

[121] Krill D., DeFlavia P., Dhir R., Luo J., Becich M.J., Lehman E., Getzenberg R.H. (2001). Expression patterns of vitamin D receptor in human prostate. J. Cell. Biochem..

[122] Schwartz G.G. (2005). Vitamin D and the epidemiology of prostate cancer. Semin. Dial..

[123] Galor O., Moav O., The Neolithic Revolution and Contemporary Variations in Life Expectancy, Working Paper, No 2007-14; Brown University, Department of Economics, 2007, Providence, RI. https://www.econstor.eu/handle/10419/80105.

[124] Bell K.J.L., Del Mar C., Wright G., Dickinson J., Glasziou P. (2015). Prevalence of incidental prostate cancer: A systematic review of autopsy studies. Int. J. Cancer.

[125] Tzenios N., Tazanios M.E., Chahine M. (2022). The impact of body mass index on prostate cancer: An updated systematic review and meta-analysis. Medicine.

[126] Wright M.E., Chang S.C., Schatzkin A., Albanes D., Kipnis V., Mouw T., Hurwitz P., Hollenbeck A., Leitzmann M.F. (2007). Prospective study of adiposity and weight change in relation to prostate cancer incidence and mortality. Cancer.

[127] Zhong S., Yan X., Wu Y., Zhang X., Chen L., Tang J., Zhao J. (2016). Body mass index and mortality in prostate cancer patients: A dose-response meta-analysis. Prostate Cancer Prostatic Dis..

[128] Bonn S.E., Wiklund F., Sjölander A., Szulkin R., Stattin P., Holmberg E., Grönberg H., Bälter K. (2014). Body mass index and weight change in men with prostate cancer: Progression and mortality. Cancer Causes Control.

[129] Bassett J.K., Severi G., Baglietto L., MacInnis R.J., Hoang H.N., Hopper J.L., English D.R., Giles G.G. (2012). Weight change and prostate cancer incidence and mortality. Int. J. Cancer.

[130] Buschemeyer W.C., Freedland S.J. (2007). Obesity and prostate cancer: Epidemiology and clinical implications. Eur. Urol..

[131] Peterson G., Barnes S. (1993). Genistein and biochanin A inhibit the growth of human prostate cancer cells but not epidermal growth factor receptor tyrosine autophosphorylation. Prostate.

[132] Davis J.N., Singh B., Bhuiyan M., Sarkar F.H. (1998). Genistein-induced upregulation of p21WAF1, downregulation of cyclin B, and induction of apoptosis in prostate cancer cells. Nutr. Cancer.

[133] Geller J., Sionit L., Partido C., Li L., Tan X., Youngkin T., Nachtsheim D., Hoffman R.M. (1998). Genistein inhibits the growth of human-patient bph and prostate cancer in histoculture. Prostate.

[134] Ji X., Liu K., Li Q., Shen Q., Han F., Ye Q., Zheng C. (2022). A mini-review of flavone isomers apigenin and genistein in prostate cancer treatment. Front. Pharmacol..

[135] Kyle E., Neckers L., Takimoto C., Curt G., Bergan R. (1997). Genistein-induced apoptosis of prostate cancer cells is preceded by a specific decrease in focal adhesion kinase activity. Mol. Pharmacol..

[136] Santibáñez J.F., Navarro A., Martínez J. (1997). Genistein inhibits proliferation and in vitro invasive potential of human prostatic cancer cell lines. Anticancer Res..

[137] Chae H.S., Xu R., Won J.Y., Chin Y.W., Yim H. (2019). Molecular targets of genistein and its related flavonoids to exert anticancer effects. Int. J. Mol. Sci..

[138] Zhang L.L., Li L., Wu D.P., Fan J.H., Li X., Wu K.J., Wang X.Y., He D.L. (2008). A novel anti-cancer effect of genistein: Reversal of epithelial mesenchymal transition in prostate cancer cells. Acta Pharmacol. Sin..

[139] Rao A., Woodruff R.D., Wade W.N., Kute T.E., Cramer S.D. (2002). Genistein and vitamin D synergistically inhibit human prostatic epithelial cell growth. J. Nutr..

[140] Yu L., Blackburn G.L., Zhou J.-R. (2003). Genistein and daidzein downregulate prostate androgen-regulated transcript-1 (PART-1) gene expression induced by dihydrotestosterone in human prostate LNCaP cancer cells. J. Nutr..

[141] Shenouda N.S., Zhou C., Browning J.D., Ansell P.J., Sakla M.S., Lubahn D.B., MacDonald R.S. (2004). Phytoestrogens in common herbs regulate prostate cancer cell growth in vitro. Nutr. Cancer.

[142] Shen J.C., Klein R.D., Wei Q., Guan Y., Contois J.H., Wang T.T.Y., Chang S., Hursting S.D. (2000). Low-dose genistein induces cyclin-dependent kinase inhibitors and G1 cell-cycle arrest in human prostate cancer cells. Mol. Carcinog..

[143] Ouchi H., Ishiguro H., Ikeda N., Hori M., Kubota Y., Uemura H. (2005). Genistein induces cell growth inhibition in prostate cancer through the suppression of telomerase activity. Int. J. Urol..

[144] Li Y., Ahmed F., Ali S., Philip P.A., Kucuk O., Sarkar F.H. (2005). Inactivation of nuclear factor kappaB by soy isoflavone genistein contributes to increased apoptosis induced by chemotherapeutic agents in human cancer cells. Cancer Res..

[145] Kousidou O.C., Mitropoulou T.N., Roussidis A.E., Kletsas D., Theocharis A.D., Karamanos N.K. (2005). Genistein suppresses the invasive potential of human breast cancer cells through transcriptional regulation of metalloproteinases and their tissue inhibitors. Int. J. Oncol..

[146] Bektic J., Berger A.P., Pfeil K., Dobler G., Bartsch G., Klocker H. (2004). Androgen receptor regulation by physiological concentrations of the isoflavonoid genistein in androgen-dependent LNCaP cells is mediated by estrogen receptor β. Eur. Urol..

[147] Chiyomaru T., Yamamura S., Fukuhara S., Yoshino H., Kinoshita T., Majid S., Saini S., Chang I., Tanaka Y., Enokida H. (2013). Genistein inhibits prostate cancer cell growth by targeting miR-34a and oncogenic HOTAIR. PLoS ONE.

[148] Chiyomaru T., Yamamura S., Fukuhara S., Hidaka H., Majid S., Saini S., Arora S., Deng G., Shahryari V., Chang I. (2013). Genistein up-regulates tumor suppressor microRNA-574-3p in prostate cancer. PLoS ONE.

[149] Thelen P., Wuttke W., Seidlová-Wuttke D. (2014). Phytoestrogens selective for the estrogen receptor beta exert anti-androgenic effects in castration resistant prostate cancer. J. Steroid Biochem. Mol. Biol..

[150] Bonkhoff H., Berges R. (2009). The evolving role of oestrogens and their receptors in the development and progression of prostate cancer. Eur. Urol..

[151] Pollak M. (2012). The insulin and insulin-like growth factor receptor family in neoplasia: An update. Nat. Rev. Cancer.

[152] Spagnuolo C., Russo G.L., Orhan I.E., Habtemariam S., Daglia M., Sureda A., Nabavi S.F., Devi K.P., Loizzo M.R., Tundis R. (2015). Genistein and cancer: Current status, challenges, and future directions. Adv. Nutr..

[153] Dalu A., Haskell J.F., Coward L., Lamartiniere C.A. (1998). Genistein, a component of soy, inhibits the expression of the EGF and ErbB2/Neu receptors in the rat dorsolateral prostate. Prostate.

[154] Kim H., Peterson T.G., Barnes S. (1998). Mechanisms of action of the soy isoflavone genistein: Emerging role for its effects via transforming growth factor beta signaling pathways. Am. J. Clin. Nutr..

[155] Cai L.Q., Cai J., Wu W., Zhu Y.S. (2011). 17α-Estradiol and genistein inhibit high fat diet induced prostate gene expression and prostate growth in the rat. J. Urol..

[156] Pollard M., Wolter W. (2000). Prevention of spontaneous prostate-related cancer in Lobund-Wistar rats by a soy protein isolate/isoflavone diet. Prostate.

[157] Mentor-Marcel R., Lamartiniere C.A., Eltoum I.E., Greenberg N.M., Elgavish A. (2001). Genistein in the diet reduces the incidence of poorly differentiated prostatic adenocarcinoma in transgenic mice (TRAMP). Cancer Res..

[158] Wang J., Eltoum I.E., Lamartiniere C.A. (2007). Genistein chemoprevention of prostate cancer in TRAMP mice. J. Carcinog..

[159] Schleicher R.L., Lamartiniere C.A., Zheng M., Zhang M. (1999). The inhibitory effect of genistein on the growth and metastasis of a transplantable rat accessory sex gland carcinoma. Cancer Lett..

[160] Pavese J.M., Farmer R.L., Bergan R.C. (2010). Inhibition of cancer cell invasion and metastasis by genistein. Cancer Metastasis Rev..

[161] Pavese J.M., Krishna S.N., Bergan R.C. (2014). Genistein inhibits human prostate cancer cell detachment, invasion, and metastasis. Am. J. Clin. Nutr..

[162] Fritz W.A., Wang J., Eltoum I.E., Lamartiniere C.A. (2002). Dietary genistein down-regulates androgen and estrogen receptor expression in the rat prostate. Mol. Cell. Endocrinol..

[163] Sĺusarz A., Jackson G.A., Day J.K., Shenouda N.S., Bogener J.L., Browning J.D., Fritsche K.L., MacDonald R.S., Besch-Williford C.L., Lubahn D.B. (2012). Aggressive prostate cancer is prevented in ERαKO mice and stimulated in ERβKO TRAMP mice. Endocrinology.

[164] Lu L.-J., Anderson K.E., Grady J.J., Nagamani M. (1996). Effects of soya consumption for one month on steroid hormones in premenopausal women: Implications for breast cancer risk reduction. Cancer Epidemiol. Biomarkers Prev..

[165] Busby M.G., Jeffcoat A.R., Bloedon L.A.T., Koch M.A., Black T., Dix K.J., Heizer W.D., Thomas B.F., Hill J.M., Crowell J.A. (2002). Clinical characteristics and pharmacokinetics of purified soy isoflavones: Single-dose administration to healthy men. Am. J. Clin. Nutr..

[166] Hong S.J., Kim S.I., Kwon S.M., Lee J.R., Chung B.C. (2002). Comparative study of concentration of isoflavones and lignans in plasma and prostatic tissues of normal control and benign prostatic hyperplasia. Yonsei Med. J..

[167] Adams K.F., Chen C., Newton K.M., Potter J.D., Lampe J.W. (2004). Soy isoflavones do not modulate prostate-specific antigen concentrations in older men in a randomized controlled trial. Cancer Epidemiol. Biomarkers Prev..

[168] Goetzl M.A., VanVeldhuizen P.J., Thrasher J.B. (2007). Effects of soy phytoestrogens on the prostate. Prostate Cancer Prostatic Dis..

[169] Yan L., Spitznagel E.L. (2009). Soy consumption and prostate cancer risk in men: A revisit of a meta-analysis. Am. J. Clin. Nutr..

[170] Andres S., Abraham K., Appel K.E., Lampen A. (2011). Risks and benefits of dietary isoflavones for cancer. Crit. Rev. Toxicol..

[171] Hebert J.R., Hurley T.G., Olendzki B.C., Teas J., Ma Y., Hampl J.S. (1998). Nutritional and socioeconomic factors in relation to prostate cancer mortality: A cross-national study. J. Natl. Cancer Inst..

[172] Perabo F.G.E., Von Löw E.C., Ellinger J., von Rücker A., Müller S.C., Bastian P.J. (2008). Soy isoflavone genistein in prevention and treatment of prostate cancer. Prostate Cancer Prostatic Dis..

[173] Van Die M.D., Bone K.M., Williams S.G., Pirotta M.V. (2014). Soy and soy isoflavones in prostate cancer: A systematic review and meta-analysis of randomized controlled trials. BJU Int..

[174] He J., Wang S., Zhou M., Yu W., Zhang Y., He X. (2015). Phytoestrogens and risk of prostate cancer: A meta-analysis of observational studies. World J. Surg. Oncol..

[175] Russo G.I., Di Mauro M., Regis F., Reale G., Campisi D., Marranzano M., Lo Giudice A., Solinas T., Madonia M., Cimino S. (2018). Association between dietary phytoestrogens intakes and prostate cancer risk in Sicily. Aging Male.

[176] Jarred R.A., Keikha M., Dowling C., McPherson S.J., Clare A.M., Husband A.J., Pedersen J.S., Frydenberg M., Risbridger G.P. (2002). Induction of apoptosis in low to moderate-grade human prostate carcinoma by red clover-derived dietary isoflavones. Cancer Epidemiol. Biomarkers Prev..

[177] Fischer L., Mahoney C., Jeffcoat A.R., Koch M.A., Thomas B.F., Valentine J.L., Stinchcombe T., Boan J., Crowell J.A., Zeisel S.H. (2004). Clinical characteristics and pharmacokinetics of purified soy isoflavones: Multiple-dose administration to men with prostate neoplasia. Nutr. Cancer.

[178] DeVere White R.W., Hackman R.M., Soares S.E., Beckett L.A., Li Y., Sun B. (2004). Effects of a genistein-rich extract on PSA levels in men with a history of prostate cancer. Urology.

